# Visible light mediated intermolecular [3 + 2] annulation of cyclopropylanilines with alkynes

**DOI:** 10.3762/bjoc.10.96

**Published:** 2014-04-29

**Authors:** Theresa H Nguyen, Soumitra Maity, Nan Zheng

**Affiliations:** 1 Department of Chemistry and Biochemistry, University of Arkansas, Fayetteville, Arkansas, 72701, USA

**Keywords:** [3 + 2], alkyne, annulation, cyclopropylaniline, photoredox catalysis, visible light

## Abstract

Intermolecular [3 + 2] annulation of cyclopropylanilines with alkynes is realized using visible light photoredox catalysis, yielding a variety of cyclic allylic amines in fair to good yields. This method exhibits significant group tolerance particularly with heterocycles. It can also be used to prepare complex heterocycles such as fused indolines.

## Introduction

Cyclopropanes have been used as a three-carbon synthon to prepare a diverse array of organic compounds [1–4]. The unusual reactivity, exhibited by cyclopropanes, is largely due to their inherent ring strain that makes cleavage of the C–C bonds facile [5]. A number of methods have been developed to regioselectively cleave cyclopropanes, generating synthetically useful intermediates that can be further manipulated [1–5]. For one subclass of cyclopropanes, cyclopropylamines, the requisite ring opening is often accomplished by one-electron oxidation of the parent amine. This oxidation step can be realized enzymatically [6–8], chemically [9–14], electrochemically [15–16], and photochemically [17–20]. Recently, visible light photoredox catalysis has emerged as a powerful method to manipulate the redox chemistry of organic compounds [21–26]. Amines have been used as an electron donor to reduce the excited state of photocatalysts, while they are oxidized to amine radical cations. Our group and others have taken advantage of this facile redox process and developed a number of synthetic methods that harness the synthetic potential of amine radical cations [21,27–28]. One of the reported methods from our group involves [3 + 2] annulation of cyclopropylanilines with alkenes [29]. We were intrigued by the possibility of extending this annulation method to include alkynes. The immediate benefits of using alkynes include eliminating the diastereoselectivity issue observed in the annulation of monocyclic cyclopropylanilines with alkenes and introducing an alkene functional group into the annulation product. Furthermore, the synthesis of cyclic allylic amines is non-trivial in general [30]. Herein, we report intermolecular [3 + 2] annulation of monocyclic cyclopropylanilines with alkynes under visible light photoredox conditions.

## Results and Discussion

Biphenylcyclopropylamine **1** and phenylacetylene (**2**) were chosen as the standard substrates to optimize the catalyst system for the [3 + 2] annulation with alkynes (Table 1). Similar to the annulation with alkenes [29], several reactivity patterns were observed. CH_3_NO_2_ was far superior to DMF and CH_3_CN as the solvent (Table 1; entries 1–3). Ru(bpz)_3_(PF_6_)_2_ was a more effective photocatalyst than Ru(bpy)_3_(PF_6_)_2_ (Table 1, entry 4). Air was detrimental to the annulation reaction (Table 1, entry 5). However, we noticed the annulation with alkynes was slower than with alkenes, previously reported by our group [29]. To compensate for lower reactivity of alkynes, we investigated commercially available light resources that were stronger than 13 W compact fluorescent lamps (CFLs). 13 W CFLs were used as the light source to mediate the annulation with alkenes [29]. White 18 W LEDs were found to be more effective for the annulation with alkynes, resulting in a higher yield (Table 1, entry 6). Control studies showed that both the photocatalyst and light were required, though some background reaction was observed (Table 1, entries 7 and 8).

**Table 1:** Catalyst screening.

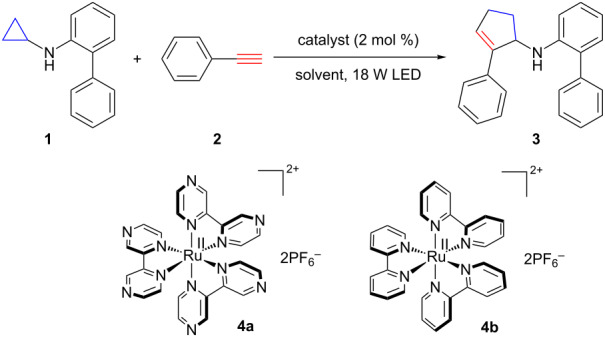				

Entry^a^	Catalyst	Light	Solvent	GC yield of **3** [%]^b^

1	**4a**	18 W LED	CH_3_NO_2_	82 (80)^c^
2	**4a**	18 W LED	DMF	20
3	**4a**	18 W LED	CH_3_CN	36
4	**4b**	18 W LED	CH_3_NO_2_	55
5^d^	**4a**	18 W LED	CH_3_NO_2_	41
6	**4a**	13 W CFL	CH_3_NO_2_	68
7	none	18 W LED	CH_3_NO_2_	6
8	**4a**	none	CH_3_NO_2_	3

^a^Conditions: **1** (0.2 mmol), **2** (1 mmol), solvent (2 mL), degassed, irradiation at rt for 8 h. ^b^Dodecane was used as an internal standard. ^c^Isolated yield by silica gel chromatography. ^d^The reaction was conducted in the presence of air.

**Figure 1:**
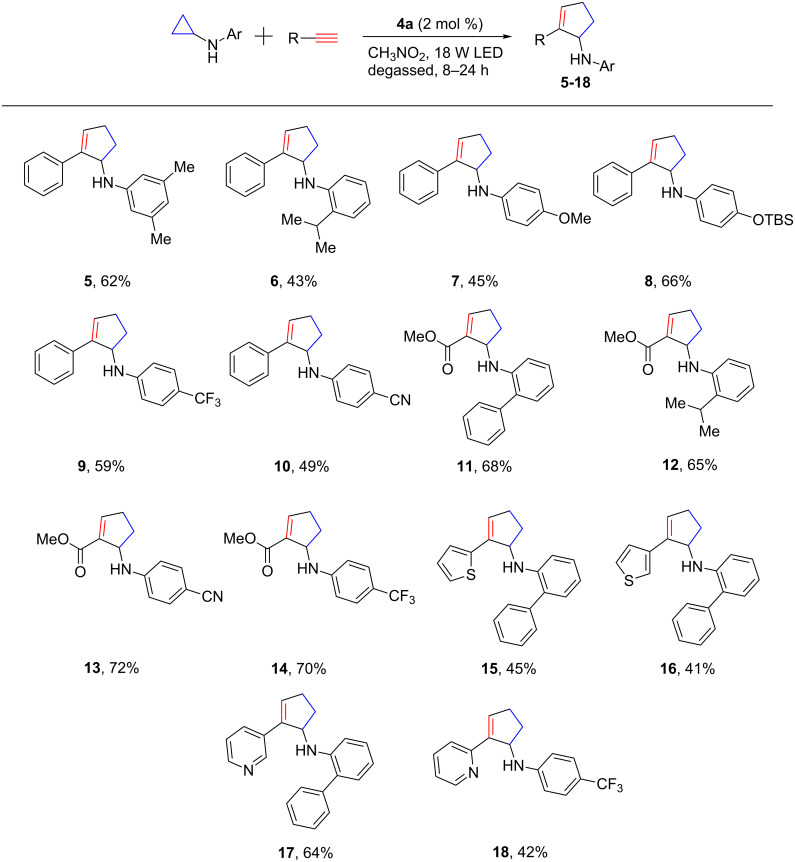
Substrate scope.

To determine the scope of this annulation process, a range of cyclopropylanilines with various electronic and steric characteristics were prepared and then subjected to the optimized catalyst system. The results of the scope studies are summarized in Figure 1. Both electron-donating (OMe, **7**, and OTBS, **8**) and electron-withdrawing (CF_3_, **9**, **14**, **18**, and CN, **10**, **13**) substituents were well tolerated, and the annulation products were generally obtained in modest to good yields. The annulation process also tolerated steric hindrance. Hindered cyclopropylanilines, such as those possessing an *ortho*-isopropyl group, were satisfactorily converted to the annulation products (**6** and **12**). With respect to the other annulation partner, terminal alkynes substituted with an electron-withdrawing group were typically required for the annulation process. Alkyl-substituted terminal alkynes and internal alkynes were not reactive under the optimized conditions. This reactivity trend towards alkynes is consistent with that exhibited in intermolecular addition of nucleophilic carbon-based radicals to alkynes [31–33]. In addition to phenylacetylene, acetylenic methyl ester is a viable annulation partner, leading to annulation products **11**–**14** in good yields. Heterocycles are frequently used in organic electronic materials [34] and pharmaceuticals [35–36]. Therefore, the ability to incorporate them is usually considered a benchmark for developing new synthetic methods. This method has certainly passed this test as two pairs of heterocycle-containing alkynes underwent the [3 + 2] annulation with cyclopropylanilines uneventfully (**15**–**18**). The alkyne moiety at the C2 or C3 position of thiophene or pyridine showed similar reactivity towards the annulation.

**Scheme 1:**
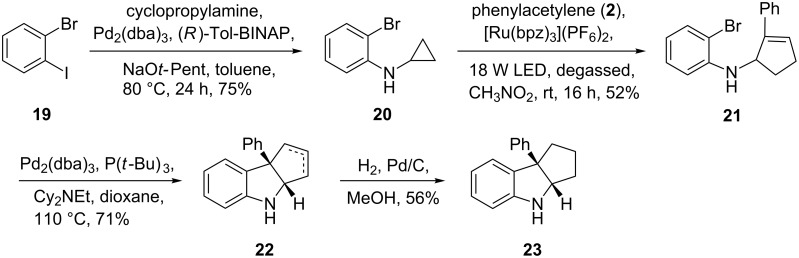
Synthesis of a fused indoline.

Fused indolines are common structural motifs that appear in a number of biologically active alkaloids and pharmaceuticals [37–38]. The [3 + 2] annulation of monocyclic cyclopropylanilines with alkynes provides a fast entry to this motif (Scheme 1). Starting from commercially available 1-bromo-2-iodobenzene (**19**) and cycloproylamine, 2-bromo-*N*-cyclopropylaniline (**20**) was prepared in 75% yield via the Buchwald–Hartwig amination [39–40]. The [3 + 2] annulation of 2-bromo-*N*-cyclopropylaniline (**20**) and phenylacetylene (**2**) was performed using the optimized catalyst system to provide cyclic allylic amine **21** in 52% yield. The fused indoline motif was formed via an intramolecular Heck reaction under Fu’s conditions [41] to provide a mixture of two olefinic regioisomers **22**, which were converted to saturated fused indoline **23** under standard catalytic hydrogenation conditions in a combined yield of 40% from **21**.

**Scheme 2:**
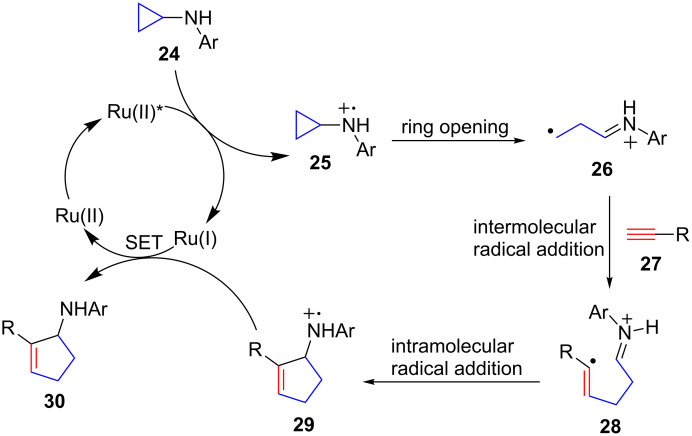
Proposed catalytic cycle.

Mechanistically, the annulation with alkynes probably proceeds through a pathway similar to the one we proposed for the annulation with alkenes (Scheme 2) [29]. The photoexcited Ru(bpz)_3_^2+^ oxidizes cyclopropylaniline **24** to the corresponding amine radical cation **25**, which triggers the cyclopropyl ring opening to generate distonic radical cation **26**. The primary carbon radical of **26** adds to the terminal carbon of alkyne **27** to afford vinyl radical **28**. Intramolecular addition of the vinyl radical to the iminium ion of distonic radical cation **28** closes the five membered ring and furnishes amine radical cation **29**. Finally, Ru(bpz)_3_^1+^ reduces amine radical cation **29** to the annulation product **30** while regenerating Ru(bpz)_3_^2+^. The proposed mechanism accounts for lower reactivity of alkynes towards intermolecular addition of nucleophilic carbon-centered radicals as well as their regiochemistry in the annulation [31–33]. Addition of radicals to alkynes generally occurs at the less hindered carbon, i.e., the terminal carbon.

## Conclusion

In summary, we have successfully expanded the [3 + 2] annulation of cyclopropylanilines to include alkynes. This annulation process with alkynes has addressed some limitations existing in the annulation with alkenes. Moreover, the annulation products from alkynes are highly useful synthetic intermediates. Their utility is demonstrated by a four-step synthesis of fused indolines in which the [3 + 2] annulation with alkynes is used to set up the backbone of indolines. Continued studies in our group will focus on further expanding the scope of the [3 + 2] annulation to include substituted anilines and other types of π-bonds.

## Experimental

General procedure for the [3 + 2] annulation of cyclopropylanilines with alkynes: an oven-dried test tube (16 × 125 mm) equipped with a stir bar was charged with [Ru(bpz)_3_](PF_6_)_2_·2H_2_O (2 mol %), cyclopropylaniline (0.2 mmol), alkyne (1.0 mmol), and dry CH_3_NO_2_ (2 mL). The test tube was sealed with a Teflon screw cap. The reaction mixture was degassed by Freeze–Pump–Thaw cycles and then irradiated at room temperature with one white LED (18 watts) positioned 8 cm from the test tube. After the reaction was complete as monitored by TLC, the mixture was diluted with diethyl ether and filtered through a short pad of silica gel. The filtrate was concentrated in vacuum and purified by silica gel flash chromatography to afford the desired allylic amine.

## Supporting Information

File 1 Experimental procedures, compound characterization, and NMR spectra.

## References

[1] Reissig H-U, Zimmer R (2003). Chem Rev.

[2] Yu M, Pagenkopf B L (2005). Tetrahedron.

[3] Carson C A, Kerr M A (2009). Chem Soc Rev.

[4] Tang P, Qin Y (2012). Synthesis.

[5] Wong H N C, Hon M-Y, Tse C-W, Yip Y-C, Tanko J, Hudlicky T (1989). Chem Rev.

[6] Zhong B, Silverman R B (1997). J Am Chem Soc.

[7] Shaffer C L, Morton M D, Hanzlik R P (2001). J Am Chem Soc.

[8] Wessjohann L A, Brandt W, Thiemann T (2003). Chem Rev.

[9] Hiyama T, Koide H, Nozaki H (1973). Tetrahedron Lett.

[10] Itoh T, Kaneda K, Teranishi S (1975). Tetrahedron Lett.

[11] Takemoto Y, Yamagata S, Furuse S, Hayase H, Echigo T, Iwata C (1998). Chem Commun.

[12] Loeppky R N, Elomari S (2000). J Org Chem.

[13] Wimalasena K, Wickman H B, Mahindaratne M P D (2001). Eur J Org Chem.

[14] Lee H B, Sung M J, Blackstock S C, Cha J K (2001). J Am Chem Soc.

[15] Li X, Grimm M L, Igarashi K, Castagnoli N, Tanko J M (2007). Chem Commun.

[16] Madelaine C, Six Y, Buriez O (2007). Angew Chem, Int Ed.

[17] Rynbrandt R H, Dutton F E (1975). J Org Chem.

[18] Lee J, Sun U J, Blackstock S C, Cha J K (1997). J Am Chem Soc.

[19] Ha J D, Lee J, Blackstock S C, Cha J K (1998). J Org Chem.

[20] Blackburn A, Bowles D M, Curran T T, Kim H (2012). Synth Commun.

[21] Prier C K, Rankic D A, MacMillan D W C (2013). Chem Rev.

[22] Xi Y, Yi H, Lei A (2013). Org Biomol Chem.

[23] Xuan J, Xiao W-J (2012). Angew Chem, Int Ed.

[24] Tucker J W, Stephenson C R J (2012). J Org Chem.

[25] Telpý F (2011). Collect Czech Chem Commun.

[26] Yoon T P, Ischay M A, Du J (2010). Nat Chem.

[27] Shi L, Xia W (2012). Chem Soc Rev.

[28] Hu J, Wang J, Nguyen T H, Zheng N (2013). Beilstein J Org Chem.

[29] Maity S, Zhu M, Shinabery R S, Zheng N (2012). Angew Chem, Int Ed.

[30] Johannsen M, Jørgensen K A (1998). Chem Rev.

[31] Fischer H, Radom L (2001). Angew Chem, Int Ed.

[32] Giese B, Lachhein S (1982). Angew Chem, Int Ed Engl.

[33] Wille U (2013). Chem Rev.

[34] Jiang W, Li Y, Wang Z (2013). Chem Soc Rev.

[35] Baumann M, Baxendale I R, Ley S V, Nikbin N (2011). Beilstein J Org Chem.

[36] Baumann M, Baxendale I R (2013). Beilstein J Org Chem.

[37] Liu D, Zhao G, Xiang L (2010). Eur J Org Chem.

[38] Xuan J, Lu L-Q, Chen J-R, Xiao W-J (2013). Eur J Org Chem.

[39] Surry D S, Buchwald S L (2011). Chem Sci.

[40] Hartwig J F (2008). Acc Chem Res.

[41] Littke A F, Fu G C (2001). J Am Chem Soc.

