# Supplementary material for: Visible light mediated intermolecular [3 + 2] annulation of cyclopropylanilines with alkynes
Source: Beilstein J Org Chem. 2014 Apr 29;10:975–80. doi: 10.3762/bjoc.10.96 (PMC4077375; doi:10.3762/bjoc.10.96)
Supplement: File 1 — Experimental procedures, compound characterization, and NMR spectra. [file Beilstein_J_Org_Chem-10-975-s001.pdf]

Supporting Information

for

Visible light mediated intermolecular [3 + 2] annulation of cyclopropylanilines with alkynes

Theresa H. Nguyen, Soumitra Maity and Nan Zheng*

Address: Department of Chemistry and Biochemistry, University of Arkansas, Fayetteville, Arkansas, 72701, USA

Email: Nan Zheng* - nzheng@uark.edu

* Corresponding author

Experimental procedures, compound characterization, and NMR spectra

Table of Contents

General considerations **S1**
General procedure 1: Preparation of *N*-cyclopropylanilines **S2**
General procedure 2: [3 + 2] annulation **S4**
Experimental procedure (intramolecular-Heck reaction) and spectroscopic data **S5**
NMR Spectra **S9**

General considerations

All reactions were carried out under a nitrogen atmosphere. Nitromethane (CH_3NO_2), acetonitrile (CH_3CN), and dimethylformamide (DMF) were pre-dried over molecular sieves. Toluene was collected under argon from a solvent purification system. Column chromatography was performed using silica gel (230–400 mesh). All new compounds were characterized by ^{1}H NMR, ^{13}C NMR, IR spectroscopy, high-resolution mass spectroscopy (HRMS), and melting point. Nuclear magnetic resonance (NMR) spectra were obtained on a Bruker Avance DPX-300 and Bruker Avance DPX-400. Chemical shifts (δ) were reported in parts per million (ppm) relative to residual proton signals in $CDCl_3$ (7.26 ppm, 77.23 ppm) at room temperature. Relative configurations of new compounds were established by HMQC experiments. IR spectra were recorded (thin film on NaCl plates) on a PerkinElmer Spectrum 100 series instrument. High

resolution mass spectra were recorded on a Bruker Ultraflex II TOF/TOF mass spectrometer. Gas chromatography/mass spectroscopy (GC/MS) analyses were performed on an Agilent 6890N Network GC System/5973 inert Mass Selective Detector. Gas chromatography analyses were performed using a Shimadzu GC-2010 Plus instrument. Melting points (m.p.) were recorded using Stuart SMP10 Melting Point Apparatus and were uncorrected.

General procedure 1: Preparation of *N*-cyclopropylanilines

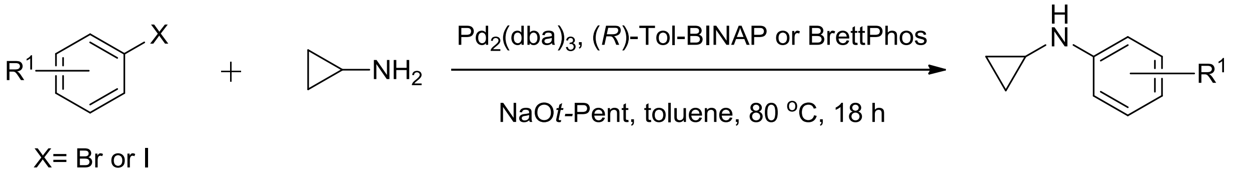

(*R*)-Tol-BINAP = (*R*)-(+)-2,2'-Bis(di-*p*-tolylphosphino)-1,1'-binaphthyl
BrettPhos = 2-(Dicyclohexylphosphino)3,6-dimethoxy-2',4',6'-triisopropyl-1,1'-biphenyl

To an oven-dried test tube equipped with a stir bar were added 0.01 mmol of $Pd_2(dba)_3$ and 0.03 mmol of ligand ((*R*)-Tol-BINAP or BrettPhos). Glove box was used to add 1.5 mmol of NaO*t*-Pent and the tube was sealed with a Teflon screw cap. 1 mmol of aromatic halide, 1.6 mmol of cyclopropylamine, and 2 mL of toluene were then added to the reaction mixture and heated at 80 °C for 18 h. After completion, the reaction mixture was cooled to room temperature, diluted with diethyl ether, filtered over a short pad of silica gel, and concentrated in vacuum. Purification by flash chromatography on silica gel afforded *N*-cyclopropylaniline.

3,5-Dimethyl-*N*-cyclopropylaniline. Following the above procedure with 5-bromo-*m*-xylene (680 µL, 5 mmol, 1 equiv) and BrettPhos (80.5 mg, 0.15 mmol, 3 mol % equiv), product was isolated after flash chromatography on silica gel (1:20 EtOAc/hexane) as a pale-yellowish oil (774 mg, 96%); IR υ_{max} (cm^{-1}) 3387, 3087, 2961, 1604, 1477, 1364, 1336, 824; ^1H NMR (400 MHz, Chloroform-*d*) δ 6.44 (dddd, *J* = 10.2, 2.9, 1.5, 0.7 Hz, 3H), 4.32 (s, 1H), 2.42 (tt, *J* = 6.4, 3.5 Hz, 1H), 2.30 – 2.20 (m, 6H), 0.76 – 0.63 (m, 2H), 0.52 (dddd, *J* = 4.9, 3.9, 3.3, 2.1 Hz, 2H); ^{13}C NMR (75 MHz, $CDCl_3$) δ 148.66, 138.68, 119.62, 110.95, 25.17, 21.43, 7.34. GC/MS *m/z* $[M+H]^+$, calc'd for $C_{11}H_{15}N$ 162; found 162.12.

***N*-Cyclopropyl-2-biphenylamine.** Following the above procedure with 2-bromobiphenyl (860 µL, 5 mmol, 1 equiv) and (*R*)-Tol-BINAP (102 mg, 0.15 mmol, 3 mol % equiv), product was isolated after flash chromatography on silica gel (1:20 EtOAc/hexane) as a colorless oil (889 mg, 85%).[1]

4-Cyano-*N*-cyclopropylaniline. Following the above procedure with 4-bromobenzonitrile (1.82 g, 10 mmol, 1 equiv) and (*R*)-Tol-BINAP (204 mg, 0.3 mmol, 3 mol % equiv), product was isolated after flash chromatography on silica gel (1:3 EtOAc/hexane) as a pale-yellowish solid

[1] Maity,S.; Zhu, M.-Z.; Shinabery, R. S.; Zheng, N. *Angew. Chem. Int. Ed.* **2012**, *51*, 222–226.

(836 mg, 53%); IR υ_{max} (cm^{-1}) 3364, 2991, 2210, 1603, 1519, 1338, 1166, 825; ^{1}H NMR (400 MHz, Chloroform-*d*) δ 7.52 – 7.39 (m, 2H), 6.79 – 6.70 (m, 2H), 4.61 (s, 1H), 2.47 (ttd, *J* = 6.6, 3.6, 0.6 Hz, 1H), 0.88 – 0.74 (m, 2H), 0.63 – 0.50 (m, 2H); ^{13}C NMR (101 MHz, CDCl3) δ 151.96, 133.53, 120.53, 112.78, 99.20, 24.60, 7.63; GC/MS *m/z* $[M+H]^+$, calc'd for $C_{10}H_{10}N_2$ 159; found 159.09.

4-Methoxy-*N*-cyclopropylaniline. Following the above procedure with 4-bromoanisole (628 μL, 5 mmol, 1 equiv) and BrettPhos (80.5 mg, 0.15 mmol, 3 mol % equiv), product was isolated after flash chromatography on silica gel (1:5 EtOAc/hexane) as a pale-yellowish oil (602 mg, 74%); IR υ_{max} (cm^{-1}) 3378, 3002, 2947, 2832, 1607, 1512, 1365, 1237, 1035, 821; ^{1}H NMR (400 MHz, Chloroform-*d*) δ 6.87 – 6.70 (m, 4H), 3.95 (d, *J* = 14.8 Hz, 1H), 3.76 (s, 3H), 2.39 (tt, *J* = 6.5, 3.6 Hz, 1H), 0.75 – 0.65 (m, 2H), 0.55 – 0.46 (m, 2H); ^{13}C NMR (101 MHz, $CDCl_3$) δ 152.34, 142.89, 114.80, 114.19, 55.85, 25.94, 7.30; GC/MS *m/z* $[M+H]^+$, calc'd for $C_{10}H_{13}NO$ 164; found 164.10.

2-Bromo-*N*-cyclopropylaniline. Following the above procedure with 1-bromo-2-iodobenzene (128 μL, 1 mmol, 1 equiv) and (*R*)-Tol-BINAP (20.4 mg, 0.03 mmol, 3 mol % equiv), product was isolated after flash chromatography on silica gel (1:150 EtOAc/hexane) as a colorless oil (160 mg, 75%).[2]

4-*tert*-Butyldimethylsilyl ether-*N*-cyclopropylaniline. Following the above procedure with (4-bromophenoxy)-*tert*-butyldimethylsilane (490 μL, 2 mmol, 1 equiv) and BrettPhos (32.2 mg, 0.06 mmol, 3 mol % equiv), product was isolated after flash chromatography on silica gel (1:5 EtOAc/hexane) as a colorless oil (486 mg, 92%); IR υ_{max} (cm^{-1}) 3377, 2945, 2858, 1509, 1465, 1364, 1249, 916, 832; ^{1}H NMR (400 MHz, Chloroform-*d*) δ 6.71 – 6.49 (m, 4H), 3.86 (s, 1H), 2.29 (tt, *J* = 6.6, 3.1 Hz, 1H), 0.94 – 0.83 (m, 9H), 0.63 – 0.52 (m, 2H), 0.44 – 0.33 (m, 2H), 0.11 – 0.02 (m, 6H); ^{13}C NMR (101 MHz, $CDCl_3$) δ 147.67, 143.11, 120.53, 114.02, 25.90, 25.79, 18.20, 7.27, -4.45; GC/MS *m/z* $[M+H]^+$, calc'd for $C_{15}H_{25}NOSi$ 264; found 264.17.

2-Isopropyl-*N*-cyclopropylaniline. Following the above procedure with 1-bromo-2-isopropylbenzene (150 μL, 1 mmol, 1 equiv) and (*R*)-Tol-BINAP (20.4 mg, 0.03 mmol, 3 mol % equiv), product was isolated after flash chromatography on silica gel (100:1 EtOAc/hexane) as a colorless oil (106 mg, 60%); IR υ_{max} (cm^{-1}) 3420, 3007, 2961, 2870, 1603, 1503, 1451, 1365, 1302, 1039, 746; ^{1}H NMR (400 MHz, Chloroform-*d*) δ 7.21 – 7.07 (m, 3H), 6.84 – 6.74 (m, 1H), 4.21 (s, 1H), 2.80 (p, *J* = 6.8 Hz, 1H), 2.45 (tt, *J* = 6.4, 3.6 Hz, 1H), 1.29 – 1.23 (m, 6H), 0.82 – 0.73 (m, 2H), 0.61 – 0.52 (m, 2H); ^{13}C NMR (101 MHz, $CDCl_3$) δ 145.40, 131.90, 126.62, 124.76, 117.73, 111.80, 27.11, 25.51, 22.52, 22.36, 7.74; GC/MS *m/z* $[M+H]^+$, calc'd for $C_{12}H_{17}N$ 176; found 176.14.

4-Trifluoromethyl-*N*-cyclopropylaniline. An oven-dried schlenk tube was charged with CuI (9.5 mg, 0.05 mmol), K_2CO_3 (276 mg, 2 mmol), proline (23 mg, 0.2 mmol), cyclopropylamine (140 μL, 2 mmol), 4-iodobenzotrifluoride (150 μL, 1 mmol), DMSO (2 mL) and a stir bar. After purging with argon for a few seconds, the tube was sealed with a Teflon screw cap. The mixture was heated at 70 °C for 12 h. The reaction mixture was then cooled to room temperature,

[2] Rousseaux, S.; Liégault, B.; Fagnou, K. *Chem Sci*. **2012**, *3*, 244–248.

quenched with brine and diluted with diethyl ether. The organic layer was separated and the aqueous layer was extracted with diethyl ether. The combined organic layers were dried over Na_2SO_4, filtered, and concentrated under vacuum. Purification of the residual mass by silica gel flash chromatography (5% EtOAc/hexane) afforded product (193 mg, 96%) as a yellowish oil.[1]

General procedure 2: [3 + 2] annulation

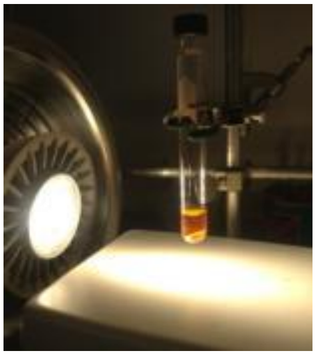

An oven-dried test tube equipped with a stir bar was charged with $[Ru(bpz)_3](PF_6)_2{\cdot}2H_2O$[3] (2 mol %), cyclopropylaniline derivative (0.2 mmol), alkyne derivative (1.0 mmol), and dry CH_3NO_2 (2 mL). The test tube was sealed with a Teflon screw cap and the reaction mixture was degassed by Freeze–Pump–Thaw cycles and irradiated at room temperature with one LED (18 watts) positioned 8 cm from the reaction vessel. After the reaction was complete, monitored by TLC, the mixture was diluted with diethyl ether and filtered through a short pad of silica gel. The solution was concentrated in vacuum and purified by silica gel flash chromatography to afford the desired cycloadducts.

Catalyst optimization

Following the above procedure, *N*-cyclopropyl-2-biphenylamine (**1**, 42 mg, 0.2 mmol), phenylacetylene (**2**, 116 µL, 1.0 mmol), $[Ru(bpz)_3](PF_6)_2{\cdot}2H_2O$[3] (**4a**, 3.8 mg, 2 mol %), and solvent (2 mL) were mixed and irradiated with a LED light for 8 h. The reaction mixture was then diluted with Et_2O (2 mL) and *n*-dodecane (45 µL) was added as the internal standard. An aliquot (0.5 mL) was filtered through a syringe filter, diluted to 1 mL, and analyzed by GC.

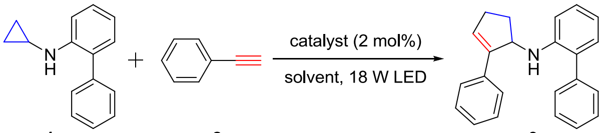

1 2 3

Entry	Conditions	Time (h)	GC Yield [%] (Isolated Yield)
**1**	**$Ru(bpz)_3^{2+}$(4a), CH_3NO_2**	**8**	**82 (80)**
2	$Ru(bpz)_3^{2+}$(**4a**), DMF	8	20
3	$Ru(bpz)_3^{2+}$(**4a**), CH_3CN	8	36
4	$Ru(bpy)_3^{2+}$(**4b**), CH_3NO_2	8	55
5	Without catalyst, CH_3NO_2	8	6
6	$Ru(bpz)_3^{2+}$(**4a**), CH_3NO_2, no LED	8	3
7	$Ru(bpz)_3^{2+}$(**4a**), CH_3NO_2, air	8	41
8	$Ru(bpz)_3^{2+}$(**4a**), CH_3NO_2, 13W CFL	8	68

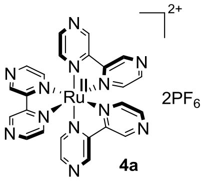

4a

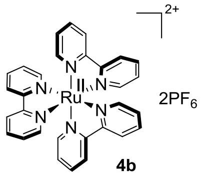

4b

[3] Rillema, D. P.; Allen, G.; Meyer, T. J.; Conrad, D. *Inorg. Chem.* **1983**, *22*, 1617–1622.

Procedure for intramolecular-Heck application

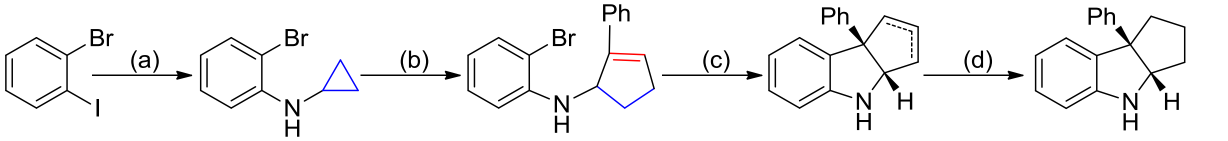

19 **20**, 75% **21**, 52% **22**, 71% **23**, 56%

Reaction conditions: (a) cyclopropylamine, $Pd_2(dba)_3$, (R)-Tol-BINAP, NaO*t*-Pent, toluene, 80 °C, 24 h; (b) phenylacetylene, $Ru(bpz)_3^{2+}$, LED, degassed, CH_3NO_2; (c) $Pd_2(dba)_3$, P(*t*-Bu)$_3$, Cy_2NEt, dioxane, 110 °C; (d) H_2, Pd/C, MeOH

General procedure 2 was performed to provide the [3 + 2] product **21**. To an oven-dried test tube equipped with a stir were added **21** (0.2 mmol) and $Pd_2(dba)_3$ (1.5 mol %). Inside the glovebox were then added Cy_2NMe (1.1 equiv), P(*t*-Bu)$_3$ (3.0 mol %), and dioxane. The reaction vessel was heated at 110 °C for 16.5 h. After completion, the reaction was quenched with diethyl ether and filtered through a short pad of silica gel. The solution was concentrated in vacuum and purified by silica gel flash chromatography (10:1 hexane/EtOAc) to afford desired product **22** (33 mg, 71% yield). *Note: Cy_2NMe and dioxane were degassed by Freeze–Pump–Thaw cycles before taken into glovebox.*

For the hydrogenation: To a clean dried 3-neck round bottom flask equipped with a stir bar was added **22** (0.1 mmol). After stirring in anhydrous MeOH (0.4 mL) for 5 min. Pd(C) (10 mol %) was added carefully under N_2 atmosphere. A balloon filled with H_2 was equipped to the flask and stirred for 22 h at room temperature. After completion, celite was added to the reaction and stirred for additional 5 min. prior to filtering through a pad of celite and washing with MeOH. The solution was concentrated in vacuum and purified by silica gel flash chromatography (10:1 hexane/EtOAc) to afford **23** (13.1 mg, 56%).

3: white yellowish solid, m.p. 113-115 °C, (48 mg, 77%). Silica gel column chromatography (15:1 hexane/EtOAc). IR υ_{max} (cm^{-1}) 3300, 2939, 1559, 11505, 1488, 1436, 1312, 1071, 755, 695; ^{1}H NMR (400 MHz, Chloroform-*d*) δ 7.37 – 7.32 (m, 2H), 7.24 – 7.19 (m, 3H), 7.19 – 7.14 (m, 2H), 7.11 (q, *J* = 1.0 Hz, 4H), 7.00 (dt, *J* = 7.4, 1.5 Hz, 1H), 6.83 – 6.76 (m, 1H), 6.70 (tt, *J* = 7.4, 1.0 Hz, 1H), 6.18 (td, *J* = 2.5, 1.1 Hz, 1H), 4.83 (d, *J* = 7.2 Hz, 1H), 4.00 (s, 1H), 2.54 – 2.38 (m, 2H), 2.36 – 2.25 (m, 1H), 1.90 (ddt, *J* = 12.8, 8.3, 3.0 Hz, 1H); ^{13}C NMR (101 MHz, $CDCl_3$) δ 144.50, 142.88, 139.28, 134.79, 130.51, 129.94, 129.16, 128.73, 128.72, 128.42, 127.73, 127.33, 126.95, 126.41, 116.70, 110.87, 59.55, 31.88, 31.08; HRMS (ESI) *m/z* [M+H]$^+$, calc'd for $C_{23}H_{21}N$ 312.1742; found 312.1708.

5: pale yellowish oil, (32.5 mg, 62%). Silica gel column chromatography (5:1 hexane/EtOAc). IR υ_{max} (cm^{-1}) 3401, 2918, 1599, 1496, 1337, 1183, 820, 757, 691; ^{1}H NMR (400 MHz, Chloroform-*d*) δ 7.45 (ddd, *J* = 6.4, 3.8, 1.6 Hz, 2H), 7.30 – 7.21 (m, 2H), 7.21 – 7.14 (m, 1H), 6.43 – 6.30 (m, 2H), 6.27 – 6.21 (m, 2H), 4.88 – 4.77 (m, 1H), 3.68 (s, 1H), 2.60 (dddd, *J* = 15.3, 7.4, 6.0, 3.6 Hz, 1H), 2.45 (ddt, *J* = 17.6, 8.9, 3.1 Hz, 1H), 2.35 – 2.25 (m, 1H), 2.23 – 2.18 (m, 6H), 1.99 (ddt, *J* = 13.4, 8.3, 2.8 Hz, 1H); ^{13}C NMR (101 MHz, $CDCl_3$) δ 147.66, 142.82, 138.99, 134.64, 129.97, 128.53, 127.31, 126.20, 119.05, 110.91, 58.91, 31.74, 30.99, 21.58; HRMS (ESI) *m/z* [M+H]$^+$, calc'd for $C_{19}H_{21}N$ 264.1737; found 264.1708.

6: yellow/orange oil, (24.1 mg, 43%). Silica gel column chromatography (25:1 hexane/EtOAc). IR υ_{max} (cm^{-1}) 3440, 2960, 1602, 1503, 1448, 1304, 1038, 745, 692; ^{1}H NMR (400 MHz,

Chloroform-*d*) δ 7.51 – 7.42 (m, 2H), 7.29 – 7.20 (m, 2H), 7.20 – 7.07 (m, 3H), 6.83 – 6.66 (m, 2H), 6.38 (ddd, *J* = 3.2, 2.4, 1.0 Hz, 1H), 4.86 (d, *J* = 7.1 Hz, 1H), 3.75 (s, 1H), 2.67 – 2.55 (m, 2H), 2.33 (ddt, *J* = 13.1, 8.9, 7.1 Hz, 1H), 1.99 (ddt, *J* = 13.4, 8.2, 2.8 Hz, 1H), 1.11 (d, *J* = 6.8 Hz, 3H), 1.00 (d, *J* = 6.8 Hz, 3H); ^{13}C NMR (101 MHz, $CDCl_3$) δ 144.26, 142.94, 134.63, 132.23, 130.07, 128.52, 127.39, 126.76, 126.25, 125.06, 116.95, 110.81, 59.28, 31.82, 31.12, 27.08, 22.28, 22.19; HRMS (ESI) *m/z* $[M+H]^+$, calc'd for $C_{20}H_{23}N$ 278.1891; found 278.1864.

7: reddish-brown oil, (23.9 mg, 45%). Silica gel column chromatography (10:1 hexane/EtOAc). IR υ_{max} (cm^{-1}) 3394, 2929, 1510, 1231, 1178, 1038, 818, 753, 693; ^{1}H NMR (400 MHz, Chloroform-*d*) δ 7.48 – 7.39 (m, 2H), 7.29 – 7.19 (m, 2H), 7.19 – 7.11 (m, 1H), 6.78 – 6.69 (m, 2H), 6.60 – 6.49 (m, 2H), 6.30 (ddd, *J* = 3.3, 2.3, 1.0 Hz, 1H), 4.81 – 4.70 (m, 1H), 3.68 (d, *J* = 0.7 Hz, 3H), 3.50 (s, 1H), 2.63 – 2.51 (m, 1H), 2.42 (ddt, *J* = 17.5, 9.1, 3.0 Hz, 1H), 2.25 (ddt, *J* = 13.1, 9.0, 7.1 Hz, 1H), 2.02 – 1.88 (m, 1H); ^{13}C NMR (101 MHz, $CDCl_3$) δ 151.89, 142.97, 142.00, 134.74, 129.87, 128.56, 127.33, 126.22, 115.02, 114.27, 59.94, 55.89, 31.60, 31.01; HRMS (ESI) *m/z* $[M+H]^+$, calc'd for $C_{18}H_{19}NO$ 266.1532; found 266.1500.

8: reddish-brown oil, (48.1 mg, 66%). Silica gel column chromatography (10:1 hexane/EtOAc). IR υ_{max} (cm^{-1}) 3400, 2929, 2856, 1508, 1250, 922, 839, 779, 756, 693; ^{1}H NMR (400 MHz, Chloroform-*d*) δ 7.50 – 7.40 (m, 2H), 7.29 – 7.19 (m, 2H), 7.20 – 7.11 (m, 1H), 6.68 – 6.60 (m, 2H), 6.51 – 6.41 (m, 2H), 6.30 (td, *J* = 2.7, 1.0 Hz, 1H), 4.72 (dt, *J* = 7.1, 2.6 Hz, 1H), 3.41 (s, 1H), 2.66 – 2.49 (m, 1H), 2.47 – 2.35 (m, 1H), 2.24 (ddt, *J* = 13.2, 9.0, 7.1 Hz, 1H), 2.00 – 1.89 (m, 1H), 0.91 (s, 9H), 0.10 (s, 6H); ^{13}C NMR (101 MHz, $CDCl_3$) δ 147.82, 143.69, 142.89, 135.40, 130.46, 129.17, 127.93, 126.86, 121.28, 114.83, 60.60, 32.23, 31.61, 26.44, 18.84, -3.78; HRMS (ESI) *m/z* $[M+H]^+$, calc'd for $C_{23}H_{31}NOSi$ 366.2247; found 366.2208.

9: yellow solid, m.p. 97-100 °C, (37.8 mg, 59%). Silica gel column chromatography (20:1 hexane/EtOAc). IR υ_{max} (cm^{-1}) 3403, 3057, 2994, 2851, 1616, 1530, 1326, 1112, 1064, 824, 755; ^{1}H NMR (400 MHz, Chloroform-*d*) δ 7.44 – 7.33 (m, 4H), 7.30 – 7.23 (m, 2H), 7.22 – 7.16 (m, 1H), 6.64 – 6.53 (m, 2H), 6.37 (t, *J* = 2.7 Hz, 1H), 4.85 (d, *J* = 7.2 Hz, 1H), 4.03 (s, 1H), 2.69 – 2.53 (m, 1H), 2.47 (ddt, *J* = 17.6, 8.8, 3.1 Hz, 1H), 2.33 (ddt, *J* = 12.9, 8.8, 7.1 Hz, 1H), 1.93 (ddt, *J* = 13.7, 8.2, 2.9 Hz, 1H); ^{13}C NMR (75 MHz, $CDCl_3$) δ 150.26, 142.48, 134.57, 130.93, 128.98, 127.90, 127.0 (q, *J* = 3.7 Hz), 126.42, 125.16 (q, *J* = 270.7 Hz), 118.8 (q, *J* = 32.4 Hz), 112.41, 59.03, 31.74, 31.34; HRMS (ESI) *m/z* $[M+H]^+$, calc'd for $C_{18}H_{16}F_3N$ 304.1299; found 304.1268.

10: white-yellow solid, m.p. 108-109 °C, (25.5 mg, 49%). Silica gel column chromatography (5:1 hexane/ EtOAc). IR υ_{max} (cm^{-1}) 3348, 2983, 2212, 1606, 1519, 1338, 1172, 824, 769; ^{1}H NMR (400 MHz, Chloroform-*d*) δ 7.35 (dq, *J* = 8.2, 1.8, 1.1 Hz, 4H), 7.23 (tt, *J* = 6.7, 1.3 Hz, 2H), 7.21 – 7.14 (m, 1H), 6.50 (dd, *J* = 9.2, 2.2 Hz, 2H), 6.42 – 6.31 (m, 1H), 4.83 (dt, *J* = 7.3, 2.5 Hz, 1H), 4.20 (s, 1H), 2.67 – 2.52 (m, 1H), 2.46 (ddt, *J* = 17.7, 8.7, 3.1 Hz, 1H), 2.31 (ddt, *J* = 13.1, 8.7, 7.1 Hz, 1H), 1.89 (ddt, *J* = 13.7, 8.2, 3.0 Hz, 1H); ^{13}C NMR (101 MHz, $CDCl_3$) δ 150.54, 141.82, 134.02, 133.78, 130.93, 128.70, 127.70, 126.04, 120.49, 112.54, 98.61, 58.58, 31.37, 31.01; HRMS (ESI) *m/z* $[M+H]^+$, calc'd for $C_{18}H_{16}N_2$ 261.1383; found 261.1347.

11: yellow oil, (42.1 mg, 68%). Silica gel column chromatography (10:1 hexane/EtOAc). IR υ_{max} (cm^{-1}) 3418, 2949, 1718, 1507, 1456, 1289, 1097, 748, 703; ^{1}H NMR (400 MHz, Chloroform-*d*)

δ 7.39 – 7.30 (m, 4H), 7.24 (dddd, J = 10.0, 5.0, 2.4, 1.2 Hz, 1H), 7.21 – 7.14 (m, 1H), 7.08 – 6.98 (m, 1H), 6.87 (td, J = 2.6, 1.2 Hz, 1H), 6.77 – 6.66 (m, 2H), 4.61 (d, J = 7.5 Hz, 1H), 4.09 (s, 1H), 3.69 – 3.57 (m, 3H), 2.58 – 2.42 (m, 1H), 2.42 – 2.21 (m, 2H), 1.89 (dddd, J = 13.3, 6.9, 3.0, 1.7 Hz, 1H); ^{13}C NMR (101 MHz, $CDCl_3$) δ 164.83, 148.12, 144.65, 139.56, 136.59, 130.38, 129.33, 128.81, 128.59, 128.17, 127.12, 117.15, 111.52, 58.77, 51.50, 32.07, 31.21; HRMS (ESI) *m/z* $[M+H]^+$, calc'd for $C_{19}H_{19}O_2$ 294.1499; found 294.1449.

12: orange-brown oil, (33.6 mg, 65%). Silica gel column chromatography (10:1 hexane/EtOAc). IR υ_{max} (cm^{-1}) 3142, 2961, 1717, 1602, 1504, 1448, 1361, 1291, 1100, 744; ^{1}H NMR (400 MHz, Chloroform-*d*) δ 7.23 – 7.14 (m, 2H), 7.14 – 7.08 (m, 1H), 6.86 – 6.70 (m, 2H), 4.78 – 4.66 (m, 1H), 4.15 (s, 1H), 3.77 (s, 3H), 2.87 (hept, J = 6.8 Hz, 1H), 2.76 – 2.62 (m, 1H), 2.62 – 2.47 (m, 1H), 2.41 (dddd, J = 13.4, 9.1, 7.4, 6.5 Hz, 1H), 2.11 – 1.98 (m, 1H), 1.26 (t, J = 6.7 Hz, 6H); ^{13}C NMR (101 MHz, $CDCl_3$) δ 165.19, 148.32, 144.53, 136.61, 133.12, 126.57, 124.97, 117.66, 111.63, 58.76, 51.62, 32.04, 31.36, 27.12, 22.52, 22.16; HRMS (ESI) *m/z* $[M+H]^+$, calc'd for $C_{16}H_{21}O_2$ 260.1643; found 260.1606.

13: white-yellow solid, m.p. 80-83 °C, (34.9 mg, 72%). Silica gel column chromatography (2:1 hexane/EtOAc). IR υ_{max} (cm^{-1}) 3356, 2949, 2211, 1714, 1606, 1523, 1296, 1173, 1098, 825; ^{1}H NMR (400 MHz, Chloroform-*d*) δ 7.49 – 7.38 (m, 2H), 7.06 (td, J = 2.6, 1.0 Hz, 1H), 6.69 – 6.60 (m, 2H), 4.93 (s, 1H), 4.74 (dtd, J = 7.6, 2.6, 1.3 Hz, 1H), 3.73 (d, J = 0.7 Hz, 3H), 2.77 – 2.63 (m, 1H), 2.61 – 2.48 (m, 1H), 2.38 (dddd, J = 13.6, 9.1, 7.6, 6.5 Hz, 1H), 2.02 – 1.90 (m, 1H); ^{13}C NMR (101 MHz, $CDCl_3$) δ 164.61, 150.60, 148.49, 135.86, 133.63, 120.44, 112.94, 98.97, 57.80, 51.76, 31.59, 31.23; HRMS (ESI) *m/z* $[M+H]^+$, calc'd for $C_{14}H_{14}N_2O_2$ 243.1117; found 243.1089.

14: pale-yellow solid, m.p. 103-105 °C, (41.8 mg, 70%). Silica gel column chromatography (10:1 hexane/EtOAc). IR υ_{max} (cm^{-1}) 3386, 2954, 1710, 1616, 1533, 1328, 1298, 1105, 1063, 826; ^{1}H NMR (400 MHz, Chloroform-*d*) δ 7.45 – 7.36 (m, 2H), 7.05 (td, J = 2.4, 1.1 Hz, 1H), 6.69 – 6.60 (m, 2H), 4.77 – 4.70 (m, 1H), 4.26 (s, 1H), 3.74 (d, J = 0.8 Hz, 3H), 2.75 – 2.60 (m, 1H), 2.59 – 2.45 (m, 1H), 2.45 – 2.31 (m, 1H), 2.02 – 1.90 (m, 1H); ^{13}C NMR (75 MHz, $CDCl_3$) δ 165.12, 150.33, 148.63, 136.51, 126.85 (q, J = 3.9 Hz), 125.31 (q, J = 269.4 Hz), 119.28 (q, J = 32.5 Hz), 112.96, 58.40, 52.04, 31.93, 31.57; HRMS (ESI) *m/z* $[M+H]^+$, calc'd for $C_{14}H_{14}F_3NO_2$ 286.1044; found 286.1010.

15: yellow solid, m.p. 104-106 °C, (30 mg, 45%). Silica gel column chromatography (100:1 hexane/EtOAc). IR υ_{max} (cm^{-1}) 3418, 3055, 2926, 1506, 1488, 1436, 1310, 771, 747, 703; ^{1}H NMR (300 MHz, Chloroform-*d*) δ 7.36 – 7.27 (m, 6H), 7.19 (dd, J = 5.1, 1.3 Hz, 1H), 7.13 (dt, J = 7.5, 1.7 Hz, 1H), 7.06 (dd, J = 3.8, 1.4 Hz, 1H), 6.97 (ddd, J = 5.1, 3.5, 1.4 Hz, 1H), 6.89 (d, J = 8.2 Hz, 1H), 6.82 (tt, J = 7.4, 1.4 Hz, 1H), 6.15 (hept, J = 1.2 Hz, 1H), 4.94 – 4.82 (m, 1H), 4.16 (s, 1H), 2.66 – 2.32 (m, 3H), 2.04 – 1.88 (m, 1H); ^{13}C NMR (75 MHz, $CDCl_3$) δ 144.66, 139.61, 139.14, 137.51, 130.85, 129.64, 129.56, 129.10, 129.00, 128.25, 127.55, 127.37, 124.64 (two carbons overlap, see HMQC), 117.21, 111.40, 61.22, 32.19, 31.27; HRMS (ESI) *m/z* $[M+H]^+$, calc'd for $C_{21}H_{19}NS$ 318.1313; found 318.1272.

16: white-yellow solid, m.p. 104-106 °C, (27.2 mg, 41%). Silica gel column chromatography (100:1 hexane/EtOAc). IR υ_{max} (cm^{-1}) 3418, 3055, 2926, 1506, 1488, 1436, 1310, 771, 747, 703;

^{1}H NMR (400 MHz, Chloroform-*d*) δ 7.32 – 7.08 (m, 9H), 7.03 (dq, *J* = 7.3, 1.9 Hz, 1H), 6.84 – 6.65 (m, 2H), 6.04 (tt, *J* = 2.5, 1.1 Hz, 1H), 4.80 – 4.73 (m, 1H), 3.99 (s, 1H), 2.53 – 2.19 (m, 3H), 1.93 – 1.76 (m, 1H); ^{13}C NMR (101 MHz, $CDCl_3$) δ 144.52, 139.33, 138.37, 136.60, 130.54, 129.21, 129.14, 128.77, 128.72, 127.82, 127.02, 126.24, 125.48, 121.12, 116.76, 110.95, 60.49, 31.84, 30.84; HRMS (ESI) *m/z* $[M+H]^+$, calc'd for $C_{21}H_{19}NS$ 318.1305; found 318.1272.

17: yellow-brown solid, m.p. 113-117 °C, (41.7 mg, 64%). Silica gel column chromatography (25:1 hexane/EtOAc). IR υ_{max} (cm^{-1}) 3416, 3030, 2928, 2848, 1580, 1507, 1436, 1309, 748, 704; ^{1}H NMR (300 MHz, Chloroform-*d*) δ 8.74 (s, 1H), 8.50 (d, *J* = 4.8 Hz, 1H), 7.71 (dt, *J* = 7.8, 1.9 Hz, 1H), 7.35 – 7.21 (m, 7H), 7.11 (dd, *J* = 7.4, 1.7 Hz, 1H), 6.95 – 6.76 (m, 2H), 6.39 (dt, *J* = 2.6, 1.5 Hz, 1H), 4.98 (d, *J* = 6.2 Hz, 1H), 3.94 (s, 1H), 2.72 – 2.38 (m, 3H), 2.10 – 1.91 (m, 1H); ^{13}C NMR (101 MHz, $CDCl_3$) δ 148.23, 147.83, 144.09, 140.05, 139.16, 133.39, 131.77, 130.54, 130.52, 129.14, 128.73, 127.99, 127.08, 123.21, 117.10, 111.03, 59.33, 31.61, 31.20, 31.13; HRMS (ESI) *m/z* $[M+H]^+$, calc'd for $C_{22}H_{20}N_2$ 313.1705; found 313.1660.

18: yellow-brown solid, m.p. 115-118 °C, (25.9 mg, 42%). Silica gel column chromatography (5:1 hexane/EtOAc). IR υ_{max} (cm^{-1}) 3279, 2934, 1614, 1534, 1326, 1112, 1063, 822, 775; ^{1}H NMR (400 MHz, Chloroform-*d*) δ 8.47 (ddd, *J* = 4.8, 1.8, 0.9 Hz, 1H), 7.52 (td, *J* = 7.7, 1.9 Hz, 1H), 7.39 – 7.28 (m, 2H), 7.28 – 7.22 (m, 1H), 7.05 (ddd, *J* = 7.6, 4.8, 1.2 Hz, 1H), 6.84 – 6.74 (m, 1H), 6.65 – 6.53 (m, 2H), 4.88 (d, *J* = 7.2 Hz, 1H), 4.38 (s, 1H), 2.72 – 2.55 (m, 1H), 2.55 – 2.44 (m, 1H), 2.35 (ddt, *J* = 13.2, 9.0, 7.1 Hz, 1H), 2.02 – 1.92 (m, 1H); ^{13}C NMR (75 MHz, $CDCl_3$) δ 153.62, 150.51, 149.83, 142.96, 136.93, 136.07, 126.9 (q, *J* = 3.8 Hz), 125.3 (q, *J* = 270.5 Hz), 122.50, 121.27, 118.9 (q, *J* = 32.5 Hz), 112.77, 58.98, 31.90, 31.46; HRMS (ESI) *m/z* $[M+H]^+$, calc'd for $C_{17}H_{15}F_3N_2$ 305.1253; found 305.1221.

21: white/pale yellow solid, m.p. 78-80 °C, (32.7 mg, 52%). Silica gel column chromatography (100:1 hexane/EtOAc). IR υ_{max} (cm^{-1}) 3402, 3059, 2929, 2846, 1592, 1495, 1317, 1019, 741, 691; ^{1}H NMR (400 MHz, Chloroform-*d*) δ 7.45 – 7.38 (m, 2H), 7.33 (dd, *J* = 7.9, 1.5 Hz, 1H), 7.22 (tt, *J* = 6.8, 0.9 Hz, 2H), 7.19 – 7.11 (m, 2H), 6.73 (ddd, *J* = 8.1, 1.5, 0.6 Hz, 1H), 6.50 (ddd, *J* = 7.8, 7.3, 1.5 Hz, 1H), 6.36 (ddd, *J* = 3.2, 2.4, 1.0 Hz, 1H), 4.80 (d, *J* = 7.0 Hz, 1H), 4.36 (s, 1H), 2.70 – 2.54 (m, 1H), 2.50 – 2.38 (m, 1H), 2.28 (ddt, *J* = 13.1, 8.9, 7.1 Hz, 1H), 1.92 (tdd, *J* = 10.7, 5.4, 2.8 Hz, 1H); ^{13}C NMR (101 MHz, $CDCl_3$) δ 144.48, 142.36, 134.48, 132.60, 130.57, 128.57, 128.49, 127.47, 126.13, 117.54, 111.88, 110.03, 59.45, 31.74, 31.02; HRMS (ESI) *m/z* $[M+H]^+$, calc'd for $C_{17}H_{16}BrN$ 314.0539, 316.0520; found 314.0500, 316.0479.

23: red-brown solid, m.p. 61-64 °C, (13.1 mg, 56%). Silica gel column chromatography (10:1 hexane/EtOAc). IR υ_{max} (cm^{-1}) 3392, 3050, 2949, 16030, 1483, 740, 699, 432; ^{1}H NMR (300 MHz, Chloroform-*d*) δ 7.42 – 7.28 (m, 4H), 7.26 – 7.15 (m, 1H), 7.04 (td, *J* = 7.6, 1.3 Hz, 1H), 6.92 (ddd, *J* = 7.4, 1.3, 0.6 Hz, 1H), 6.77 – 6.60 (m, 2H), 4.38 – 4.28 (m, 1H), 2.49 – 2.30 (m, 2H), 2.03 (ddt, *J* = 13.0, 11.8, 6.4 Hz, 1H), 1.94 – 1.74 (m, 2H), 1.74 – 1.57 (m, 1H); ^{13}C NMR (75 MHz, $CDCl_3$) δ 150.83, 148.36, 135.95, 128.32, 127.61, 126.31, 125.94, 124.60, 118.64, 108.79, 71.95, 62.49, 40.77, 38.09, 25.45; HRMS (ESI) *m/z* $[M+H]^+$, calc'd for $C_{17}H_{17}N$ 314.0539, 236.1426; found 236.1395.

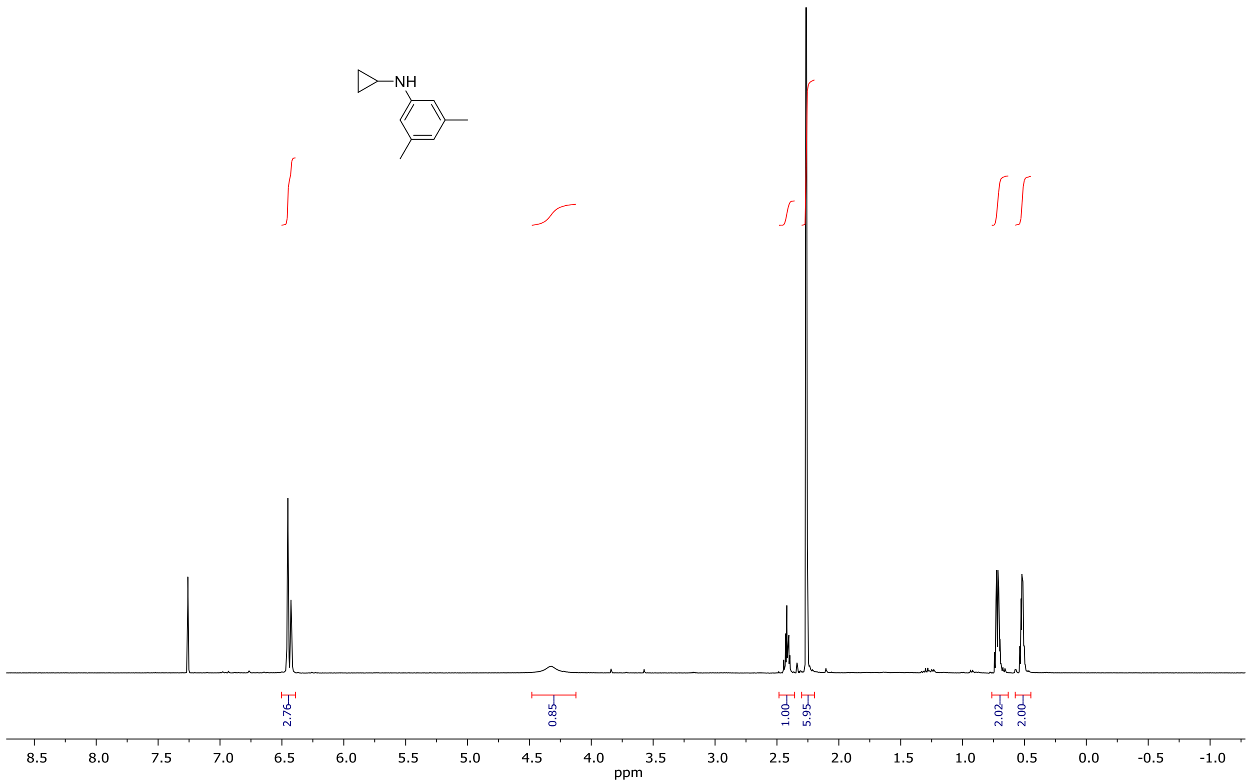
NH
2.76
0.85
1.00
5.95
2.02
2.00
8.5
8.0
7.5
7.0
6.5
6.0
5.5
5.0
4.5
4.0
3.5
3.0
2.5
2.0
1.5
1.0
0.5
0.0
-0.5
-1.0
ppm

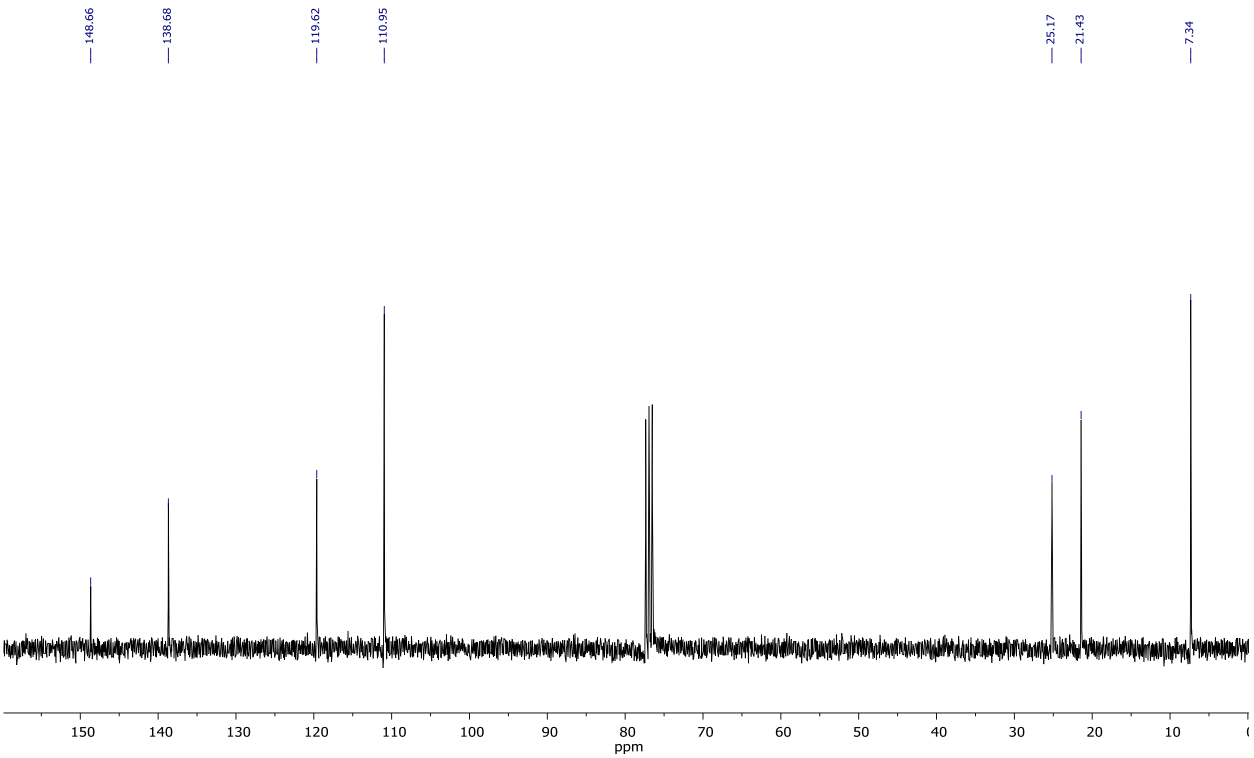
148.66
138.68
119.62
110.95
25.17
21.43
7.34
150
140
130
120
110
100
90
80
70
60
50
40
30
20
10
ppm

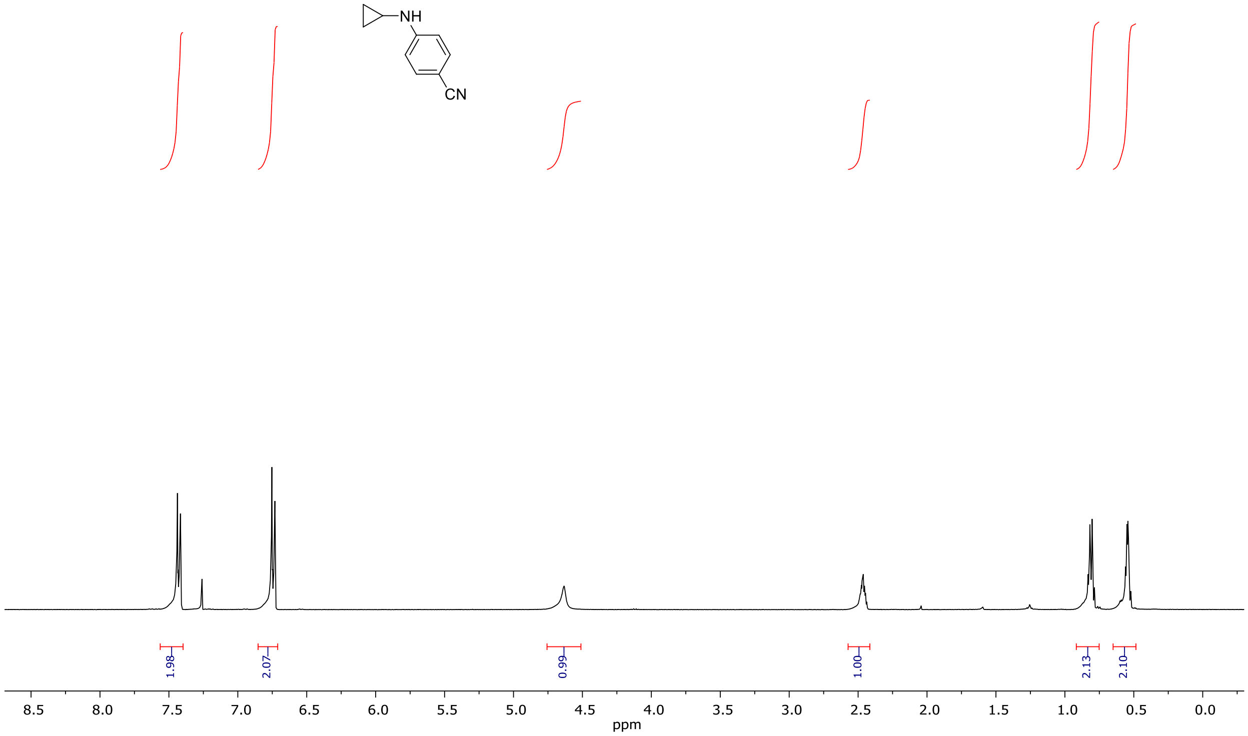
NH
CN
1.98
2.07
0.99
1.00
2.13
2.10
8.5 8.0 7.5 7.0 6.5 6.0 5.5 5.0 4.5 4.0 3.5 3.0 2.5 2.0 1.5 1.0 0.5 0.0
ppm

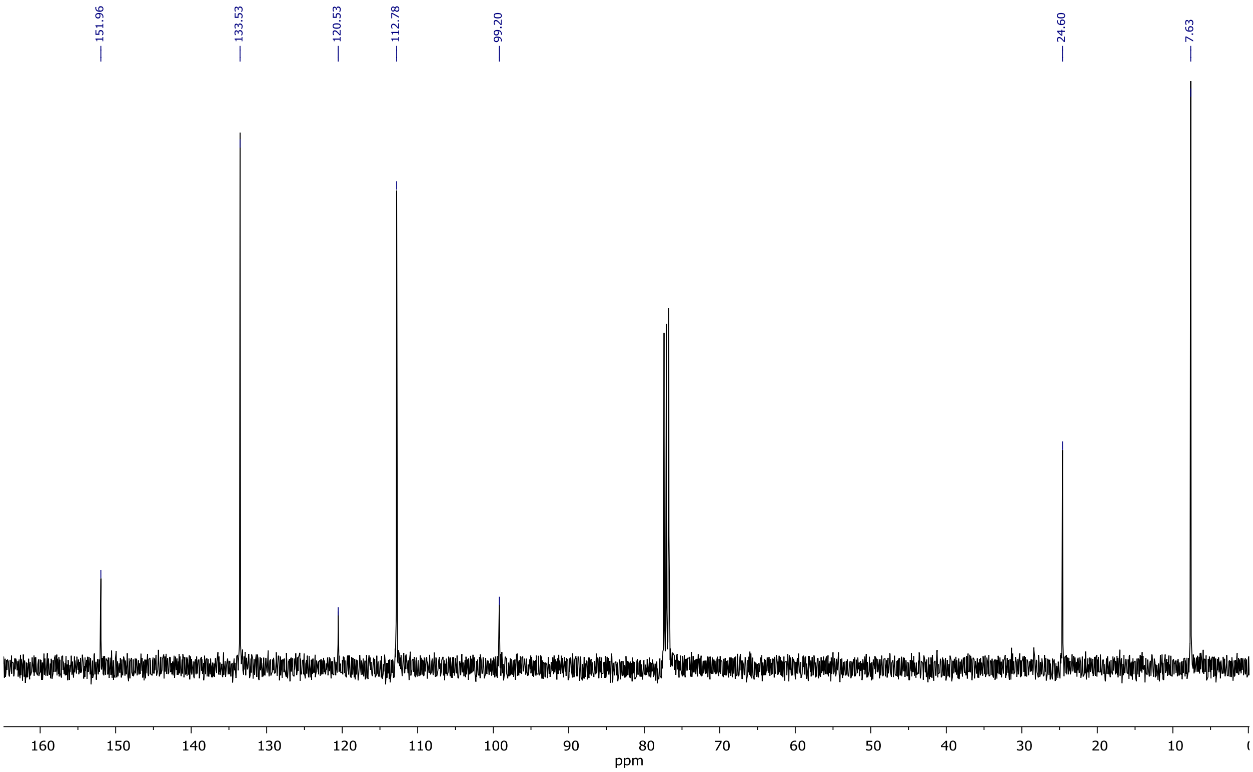
151.96
133.53
120.53
112.78
99.20
24.60
7.63
160 150 140 130 120 110 100 90 80 70 60 50 40 30 20 10
ppm

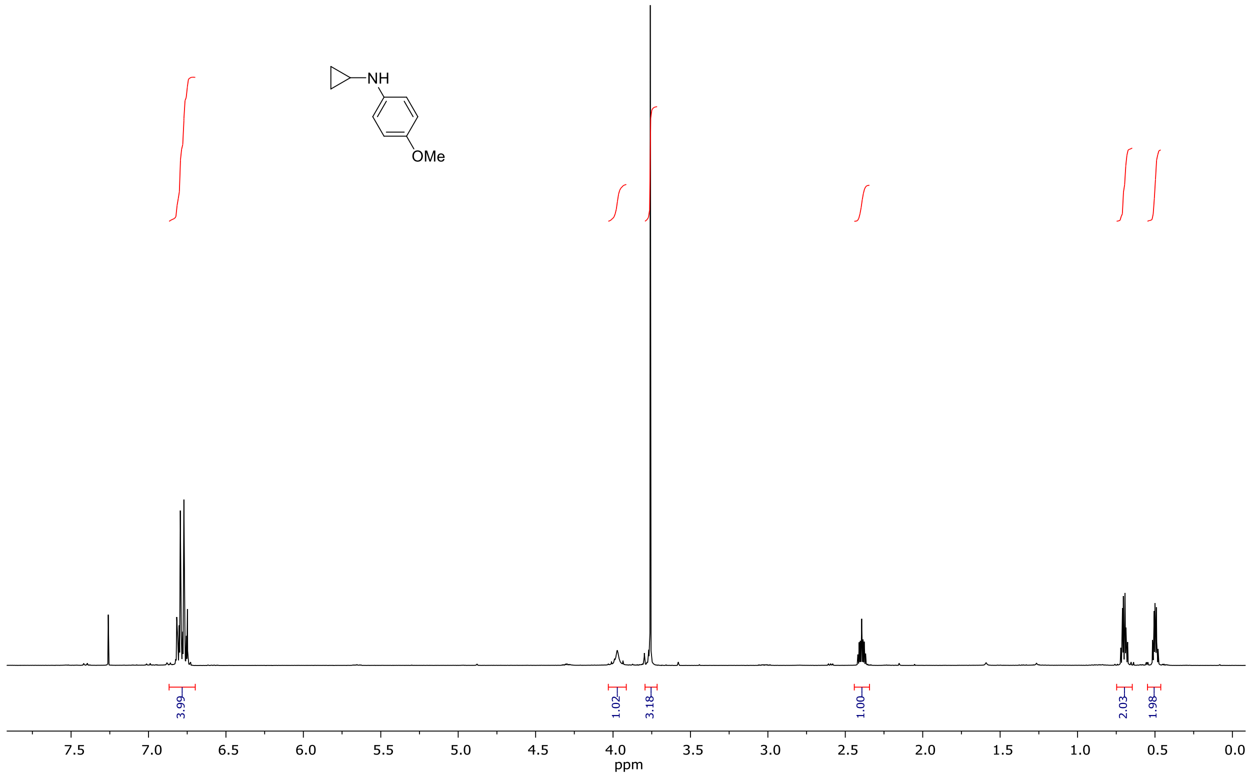
NH
OMe
3.99
1.02
3.18
1.00
2.03
1.98
7.5
7.0
6.5
6.0
5.5
5.0
4.5
4.0
3.5
3.0
2.5
2.0
1.5
1.0
0.5
0.0
ppm

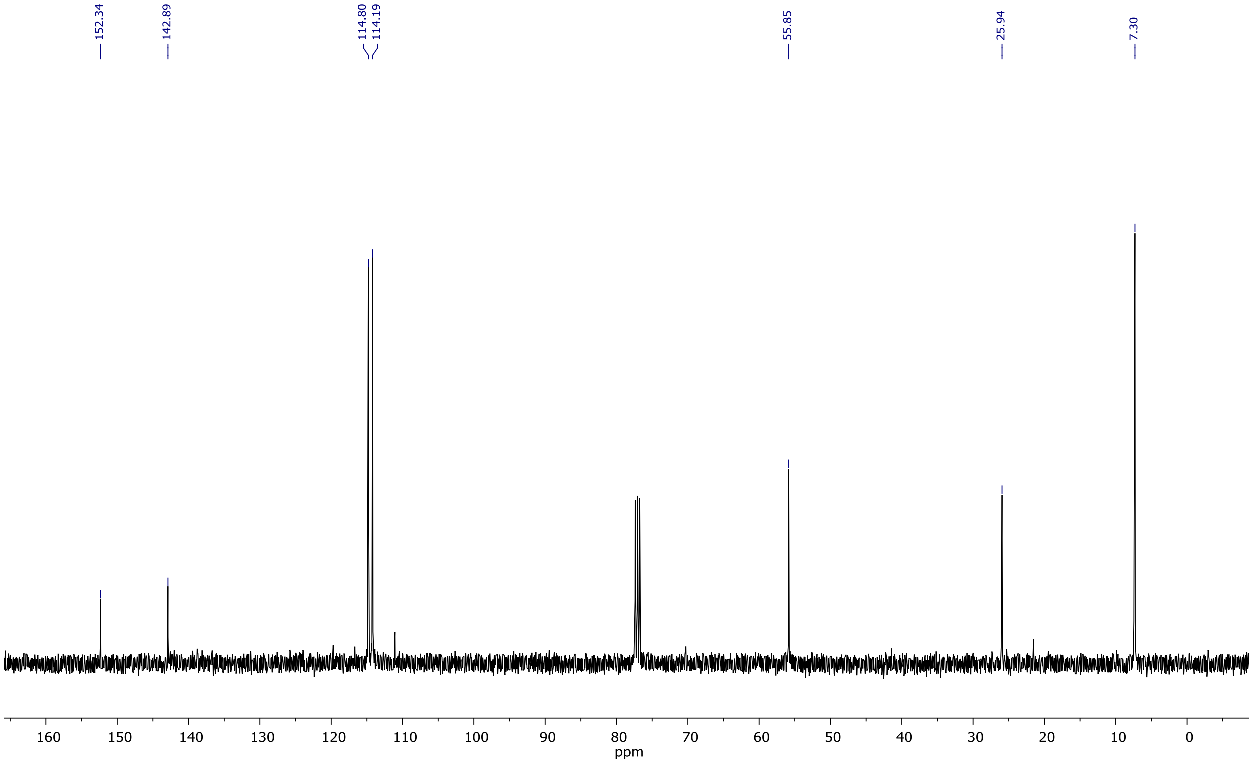
152.34
142.89
114.80
114.19
55.85
25.94
7.30
160
150
140
130
120
110
100
90
80
70
60
50
40
30
20
10
0
ppm

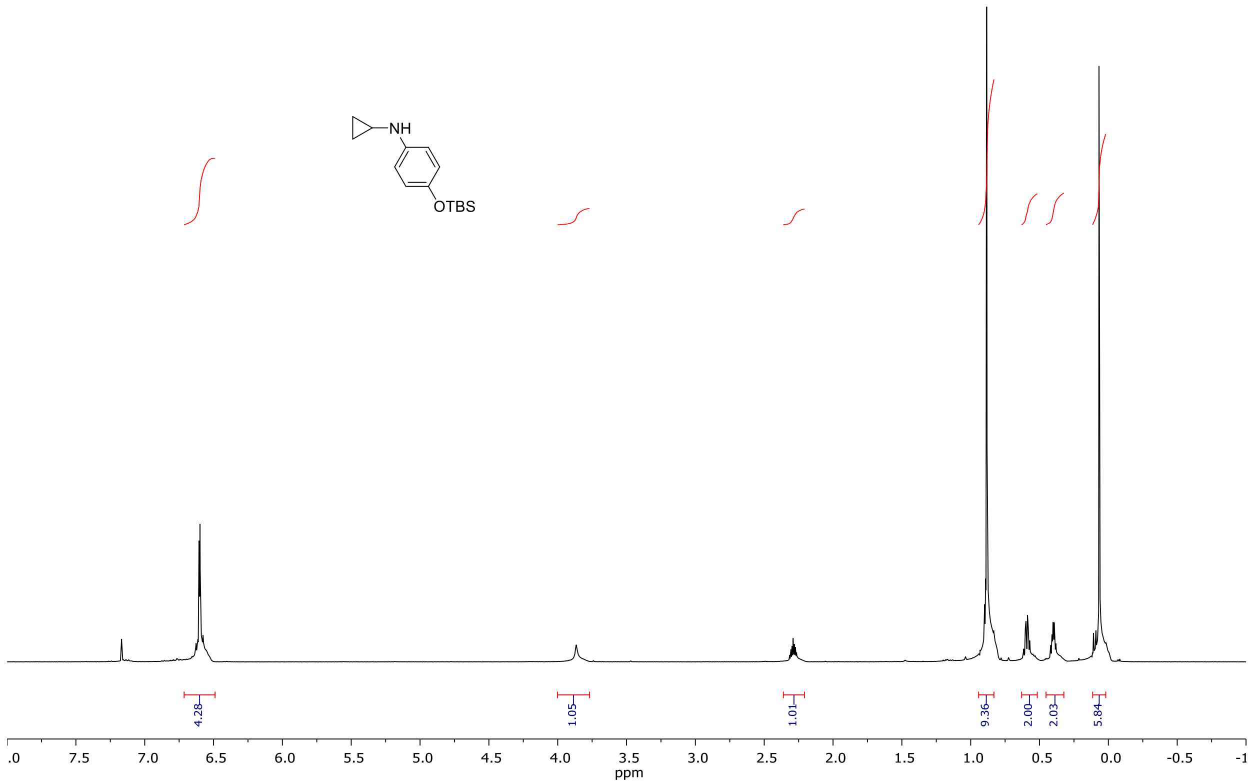

NH
OTBS
4.28
1.05
1.01
9.36
2.00
2.03
5.84
ppm

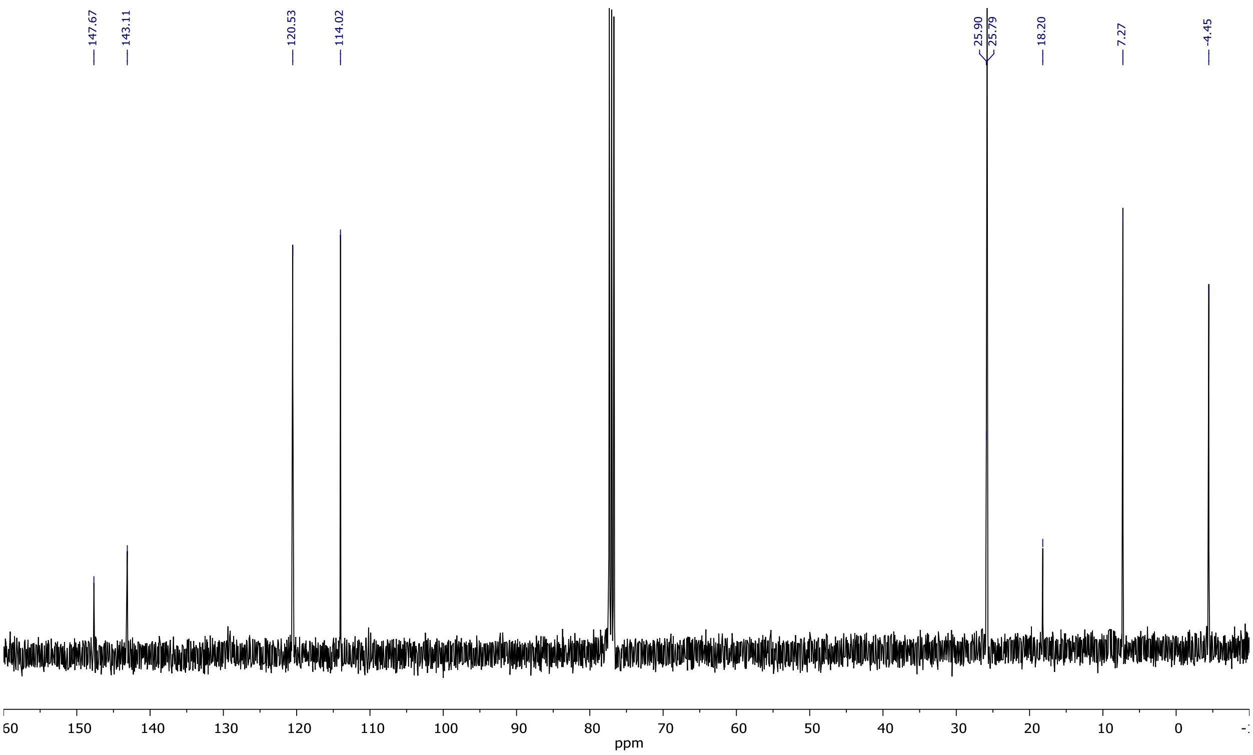

147.67
143.11
120.53
114.02
25.90
25.79
18.20
7.27
-4.45
ppm

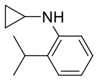
NH

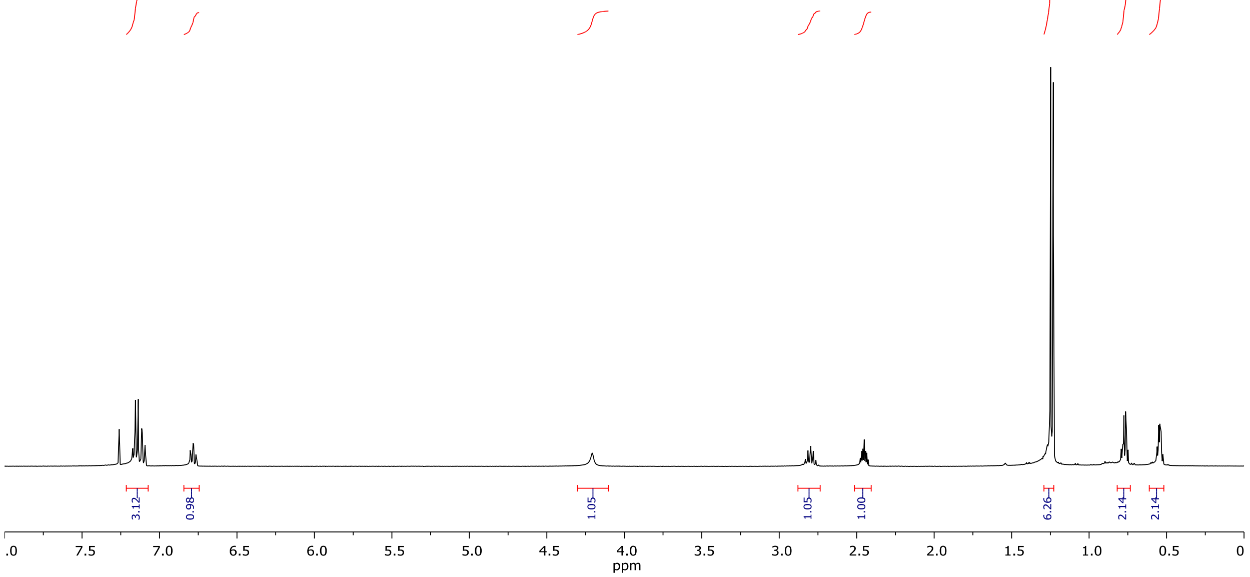
3.12
0.98
1.05
1.05
1.00
6.26
2.14
2.14
ppm

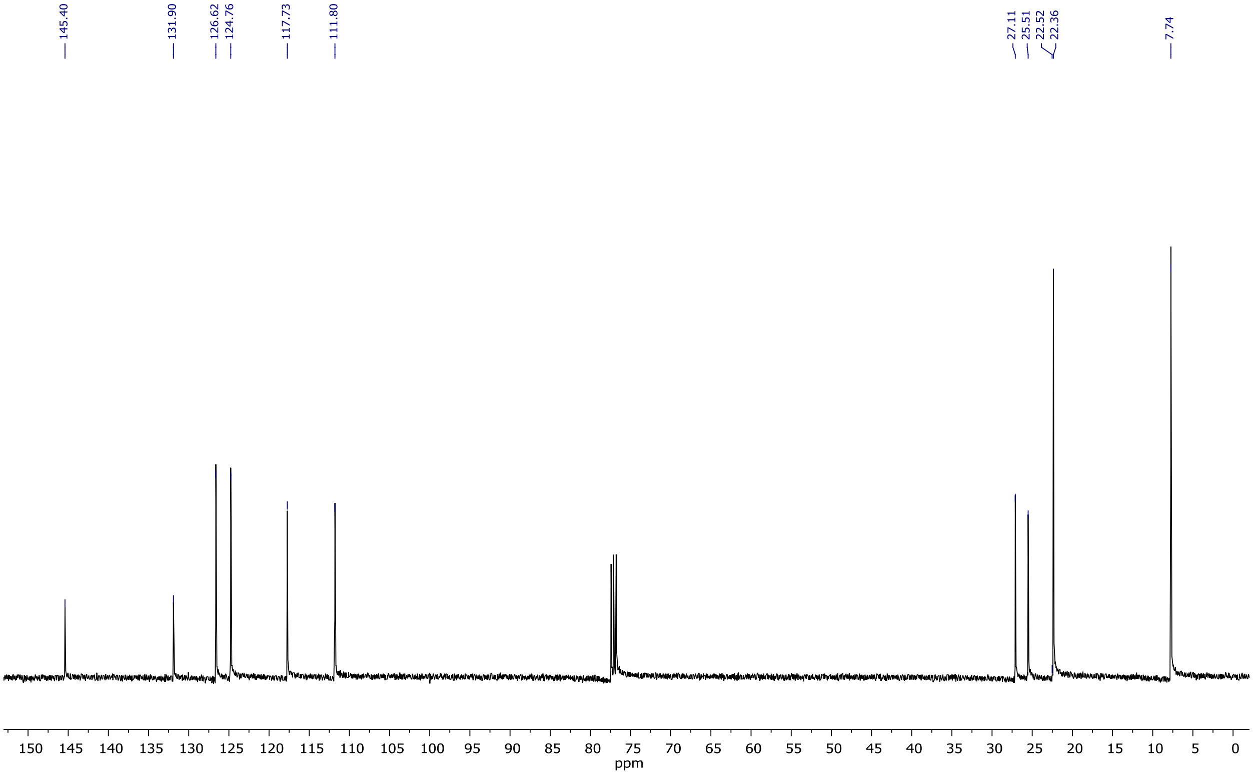
145.40
131.90
126.62
124.76
117.73
111.80
27.11
25.51
22.52
22.36
7.74
ppm

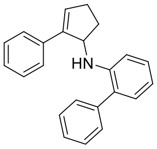
HN

3

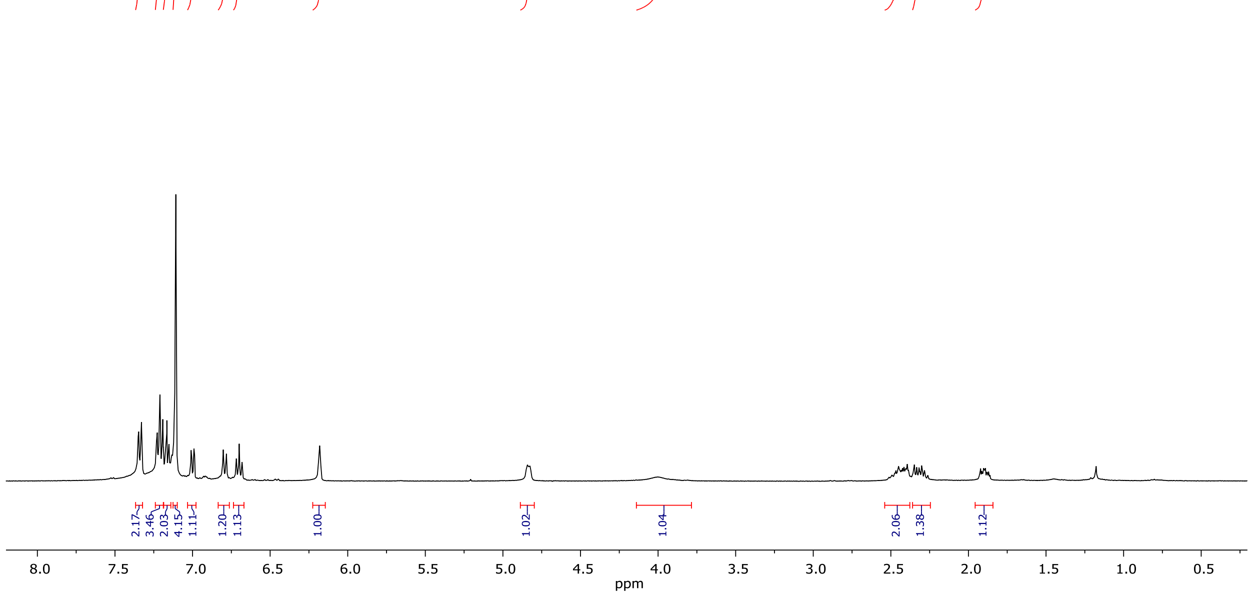
2.17
3.46
2.03
4.15
1.11
1.20
1.13
1.00
1.02
1.04
2.06
1.38
1.12
8.0 7.5 7.0 6.5 6.0 5.5 5.0 4.5 4.0 3.5 3.0 2.5 2.0 1.5 1.0 0.5
ppm

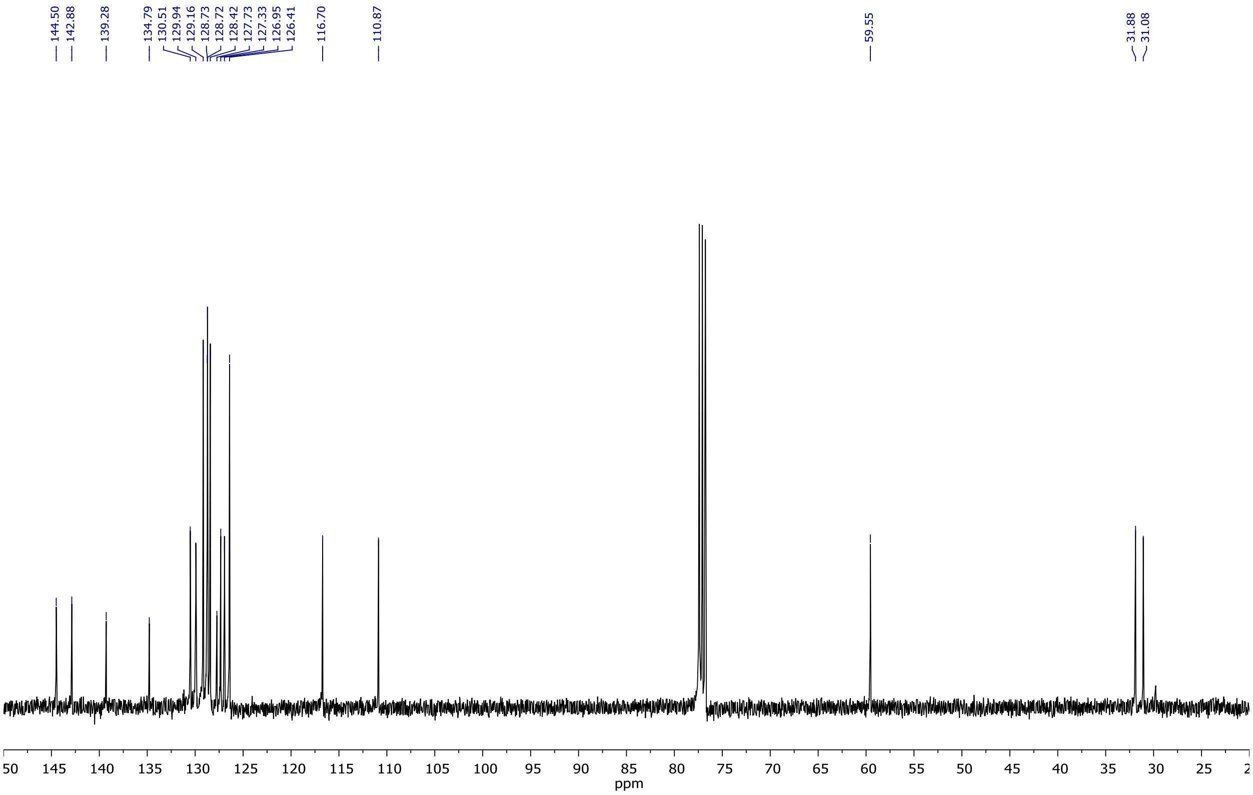
144.50
142.88
139.28
134.79
130.51
129.94
129.16
128.73
128.72
128.42
127.73
127.33
126.95
126.41
116.70
110.87
59.55
31.88
31.08
50 145 140 135 130 125 120 115 110 105 100 95 90 85 80 75 70 65 60 55 50 45 40 35 30 25
ppm

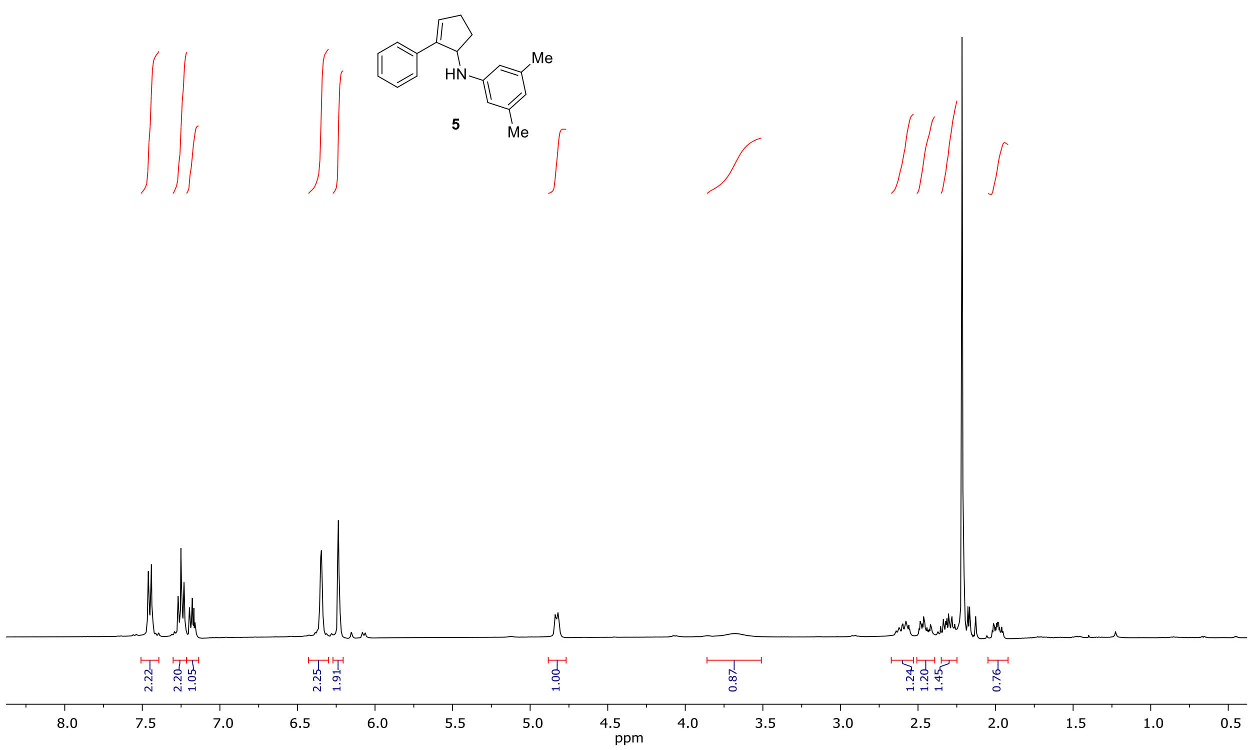
HN
Me
Me
5
2.22
2.20
1.05
2.25
1.91
1.00
0.87
1.24
1.20
1.45
0.76
8.0
7.5
7.0
6.5
6.0
5.5
5.0
4.5
4.0
3.5
3.0
2.5
2.0
1.5
1.0
0.5
ppm

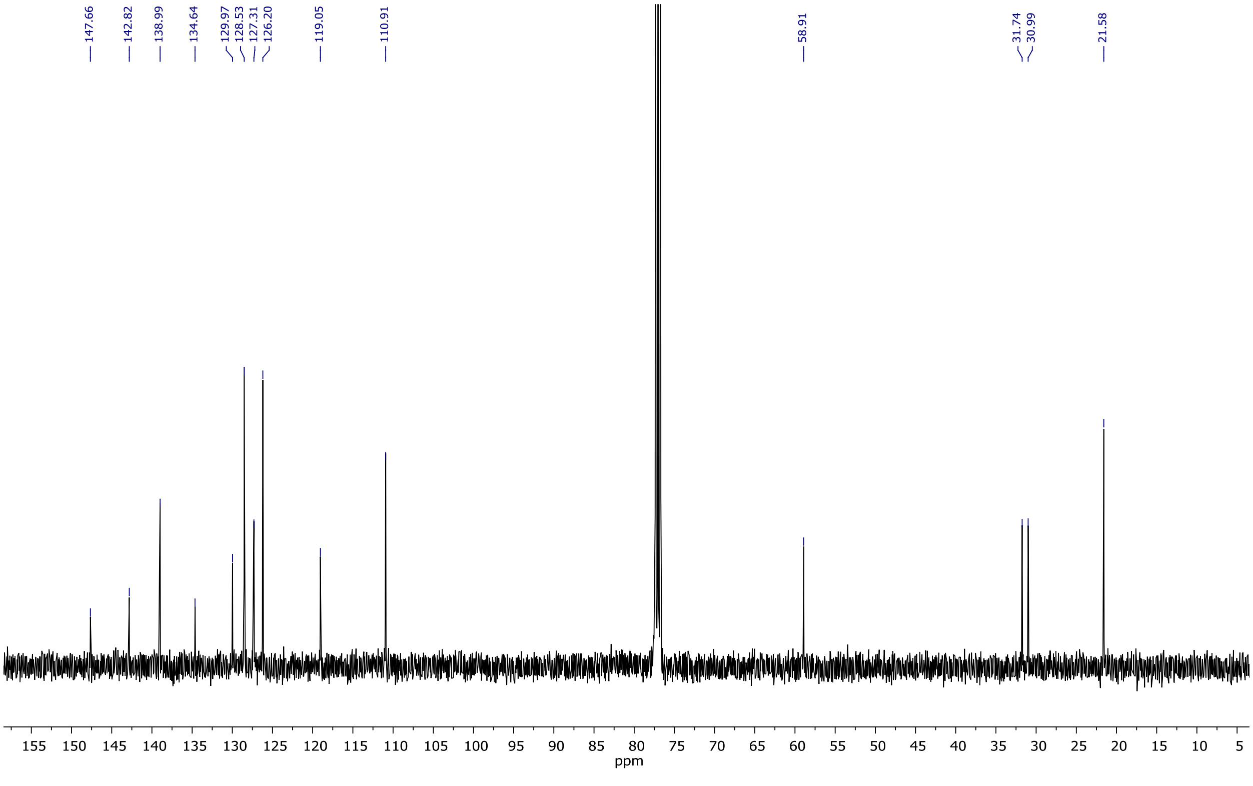
147.66
142.82
138.99
134.64
129.97
128.53
127.31
126.20
119.05
110.91
58.91
31.74
30.99
21.58
155 150 145 140 135 130 125 120 115 110 105 100 95 90 85 80 75 70 65 60 55 50 45 40 35 30 25 20 15 10 5
ppm

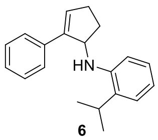

6

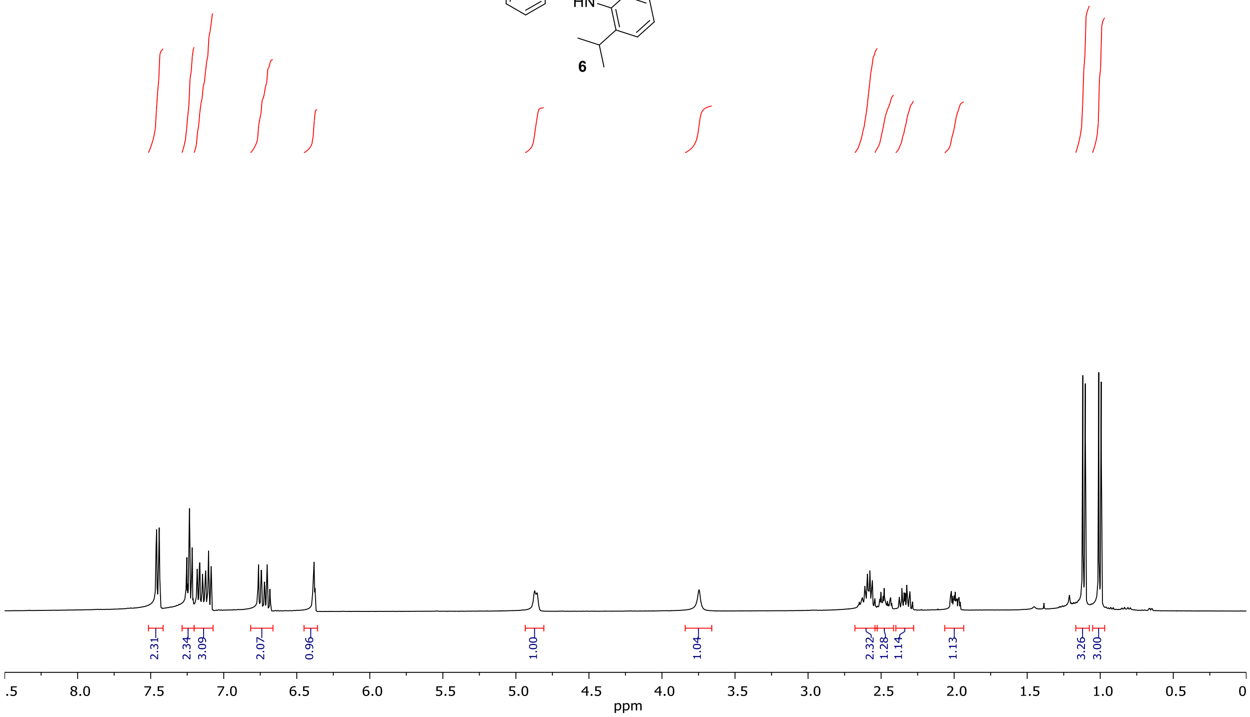
2.31
2.34
3.09
2.07
0.96
1.00
1.04
2.32
1.28
1.14
1.13
3.26
3.00
.5 8.0 7.5 7.0 6.5 6.0 5.5 5.0 4.5 4.0 3.5 3.0 2.5 2.0 1.5 1.0 0.5 0
ppm

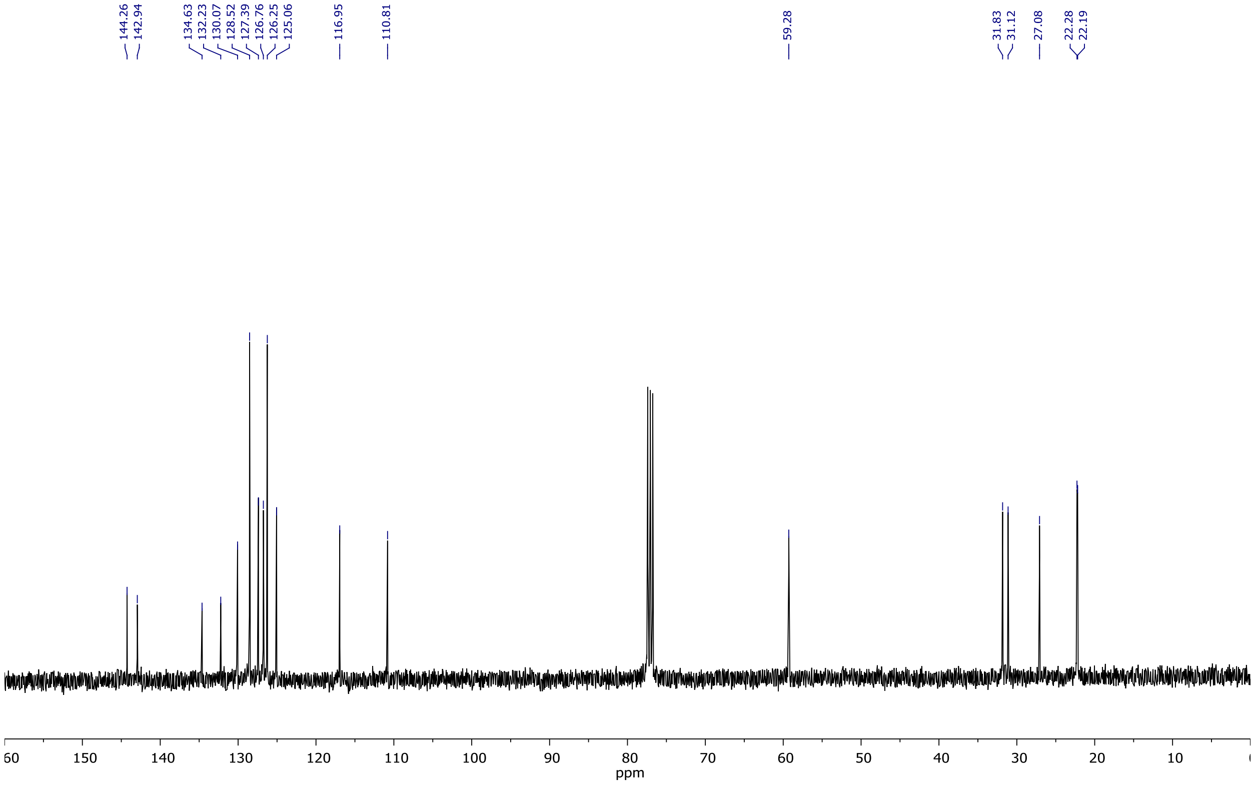
144.26
142.94
134.63
132.23
130.07
128.52
127.39
126.76
126.25
125.06
116.95
110.81
59.28
31.83
31.12
27.08
22.28
22.19
60 150 140 130 120 110 100 90 80 70 60 50 40 30 20 10
ppm

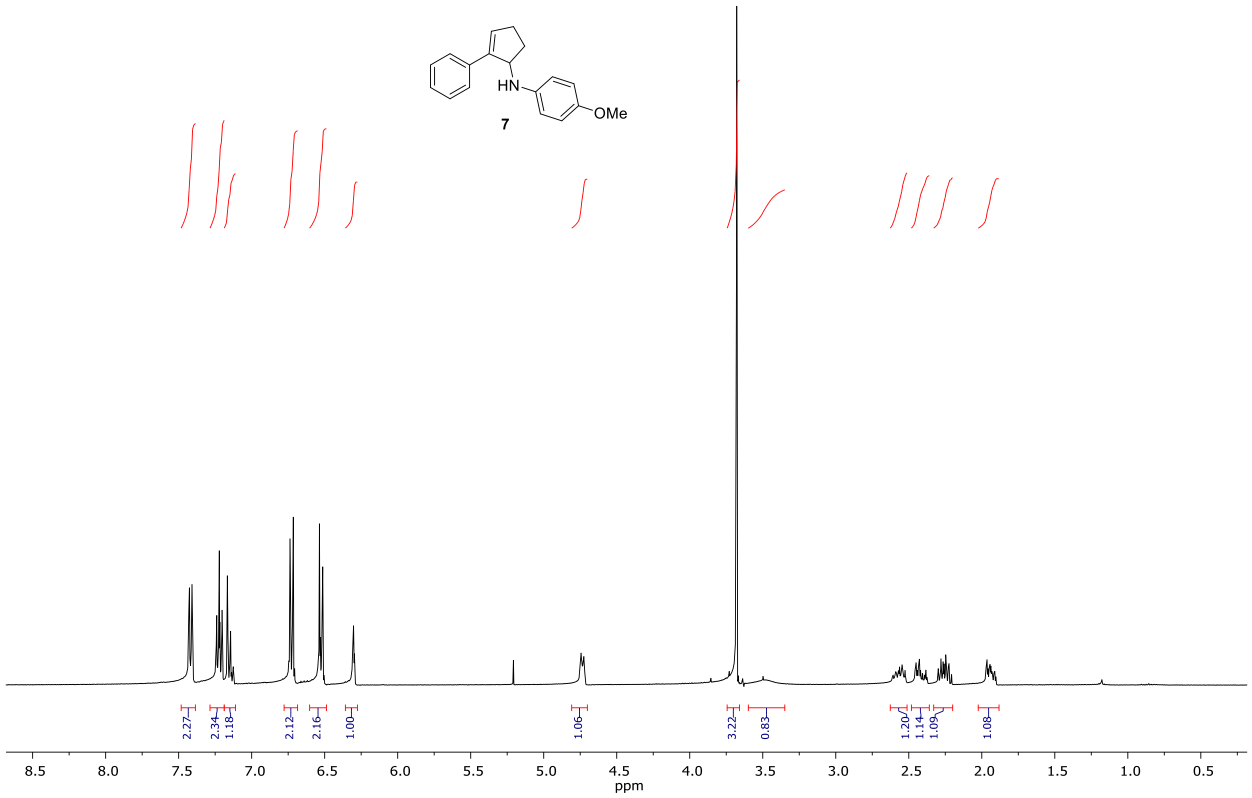
HN
OMe
7
2.27
2.34
1.18
2.12
2.16
1.00
1.06
3.22
0.83
1.20
1.14
1.09
1.08
8.5 8.0 7.5 7.0 6.5 6.0 5.5 5.0 4.5 4.0 3.5 3.0 2.5 2.0 1.5 1.0 0.5
ppm

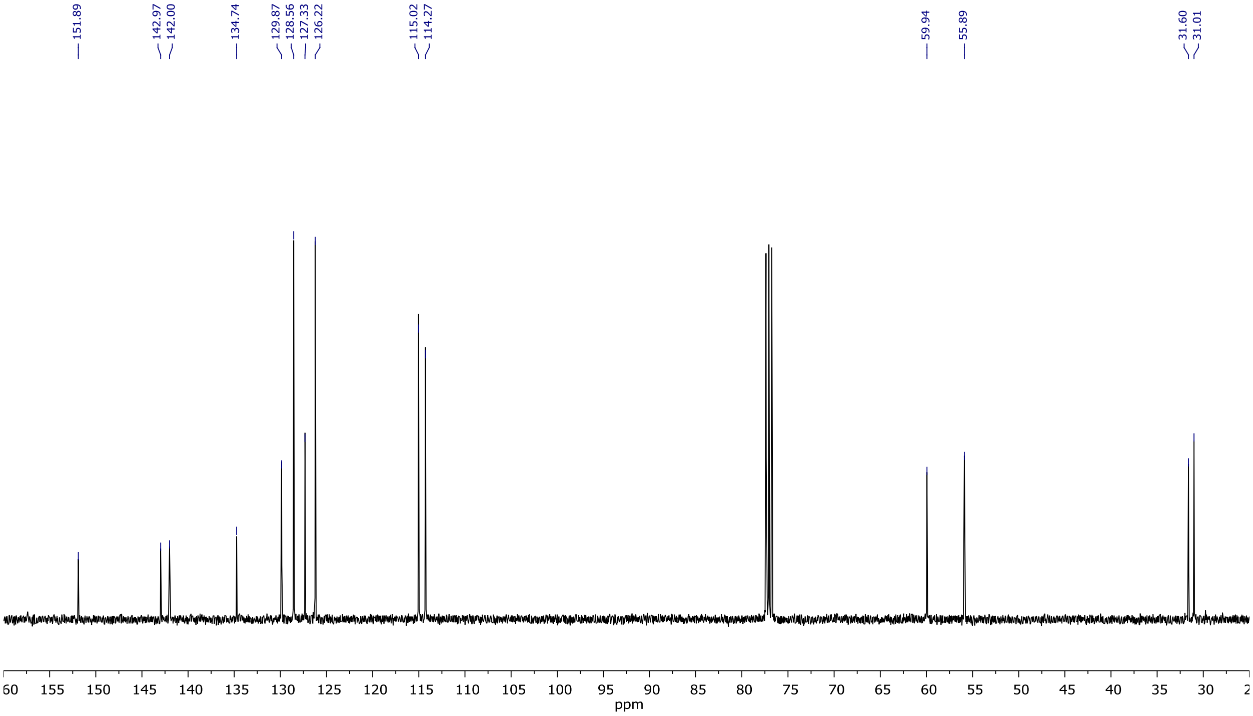
151.89
142.97
142.00
134.74
129.87
128.56
127.33
126.22
115.02
114.27
59.94
55.89
31.60
31.01
60 155 150 145 140 135 130 125 120 115 110 105 100 95 90 85 80 75 70 65 60 55 50 45 40 35 30
ppm

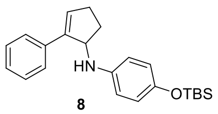
HN
OTBS

8

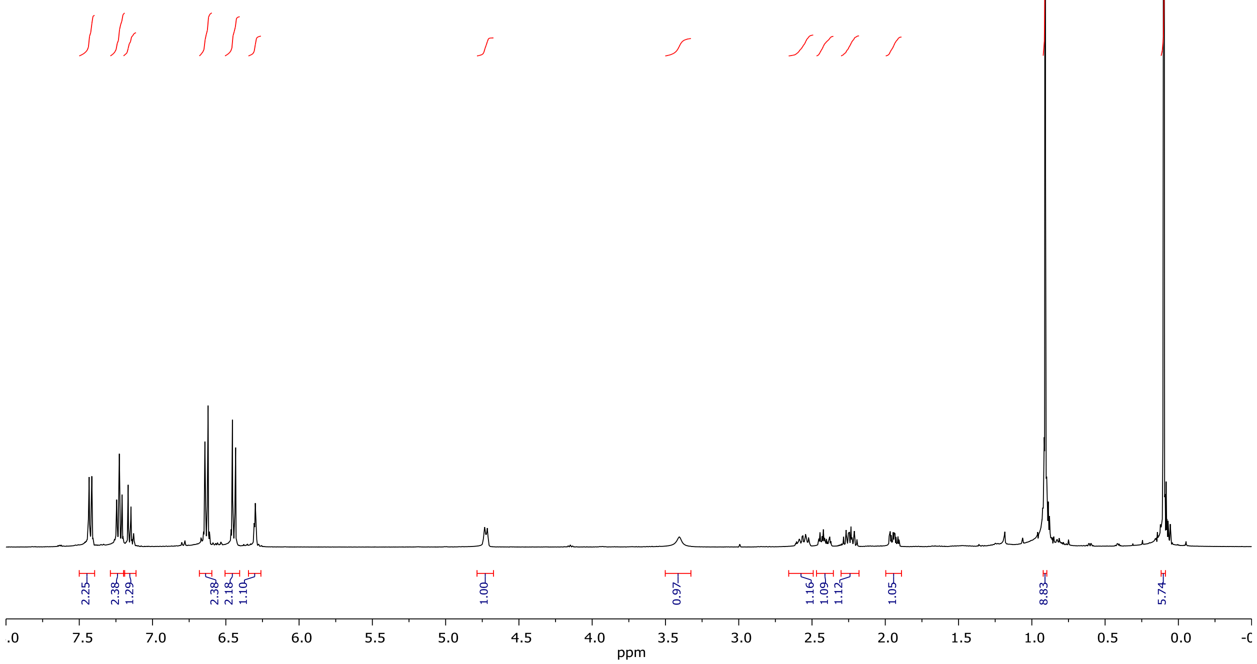
2.25
2.38
1.29
2.38
2.18
1.10
1.00
0.97
1.16
1.09
1.12
1.05
8.83
5.74
7.5 7.0 6.5 6.0 5.5 5.0 4.5 4.0 3.5 3.0 2.5 2.0 1.5 1.0 0.5 0.0
ppm

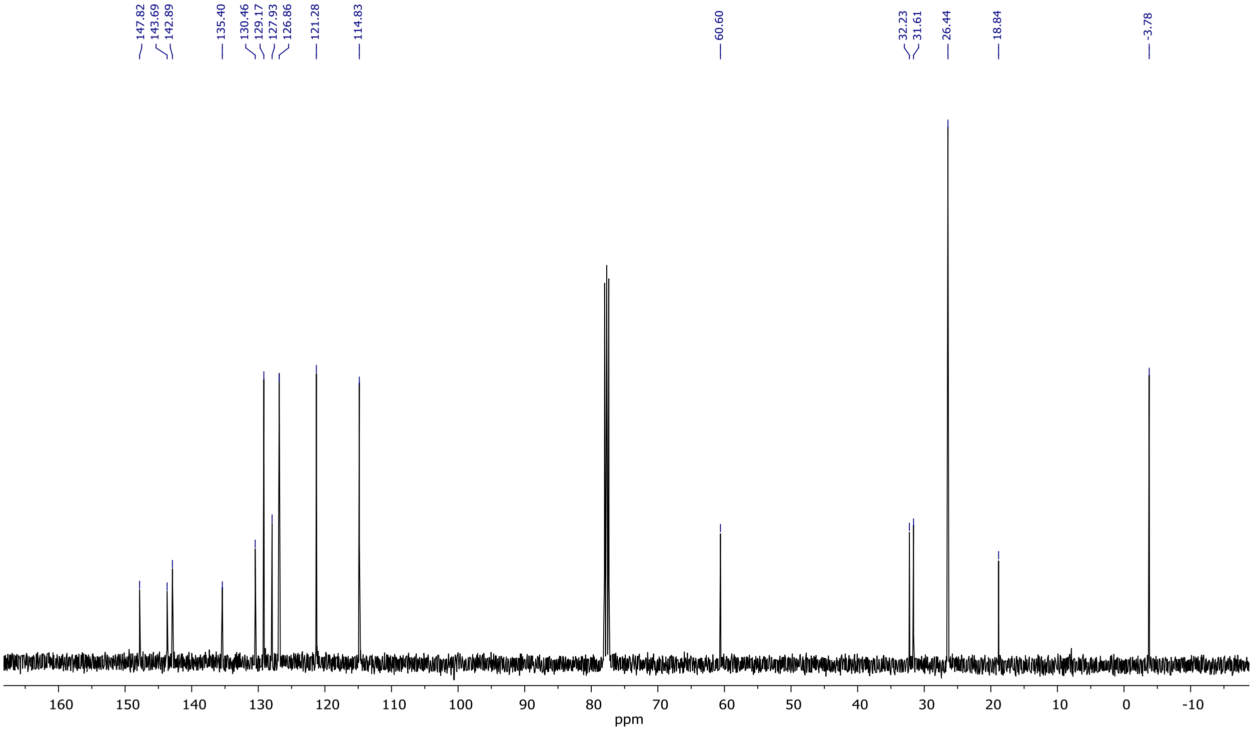
147.82
143.69
142.89
135.40
130.46
129.17
127.93
126.86
121.28
114.83
60.60
32.23
31.61
26.44
18.84
-3.78
160 150 140 130 120 110 100 90 80 70 60 50 40 30 20 10 0 -10
ppm

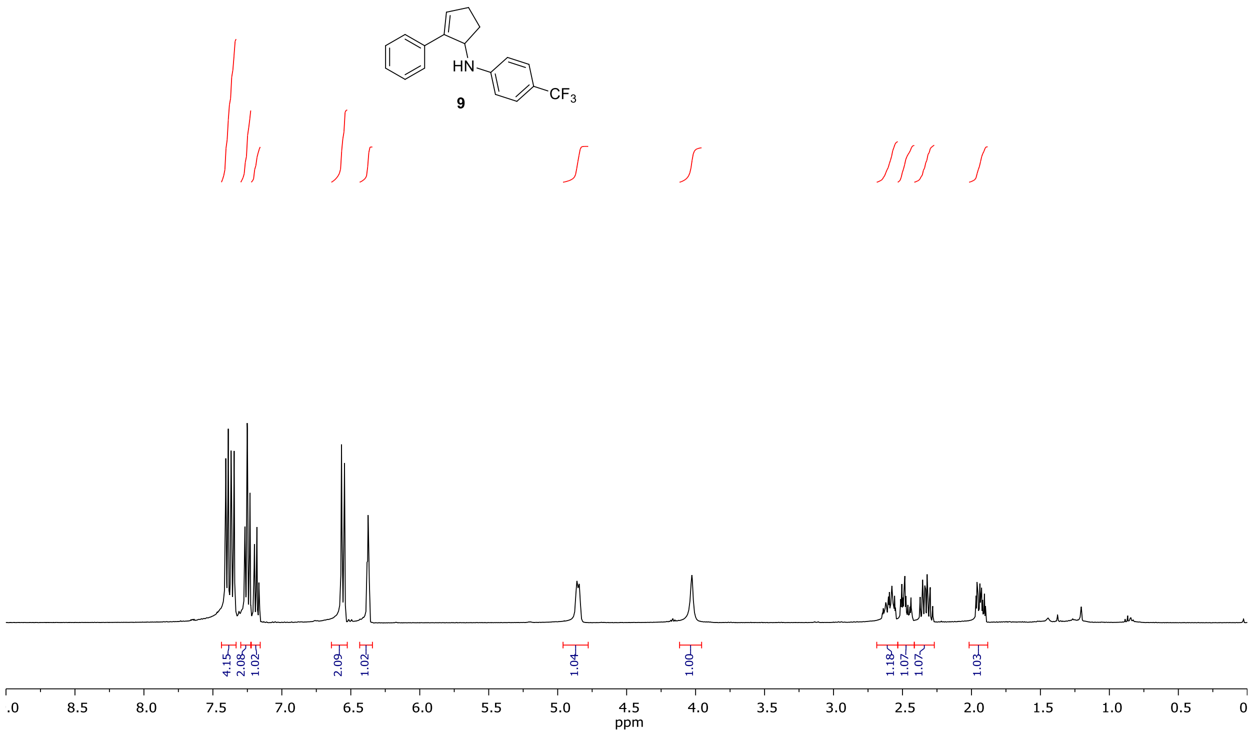
HN
CF3
9
4.15
2.08
1.02
2.09
1.02
1.04
1.00
1.18
1.07
1.07
1.03
ppm

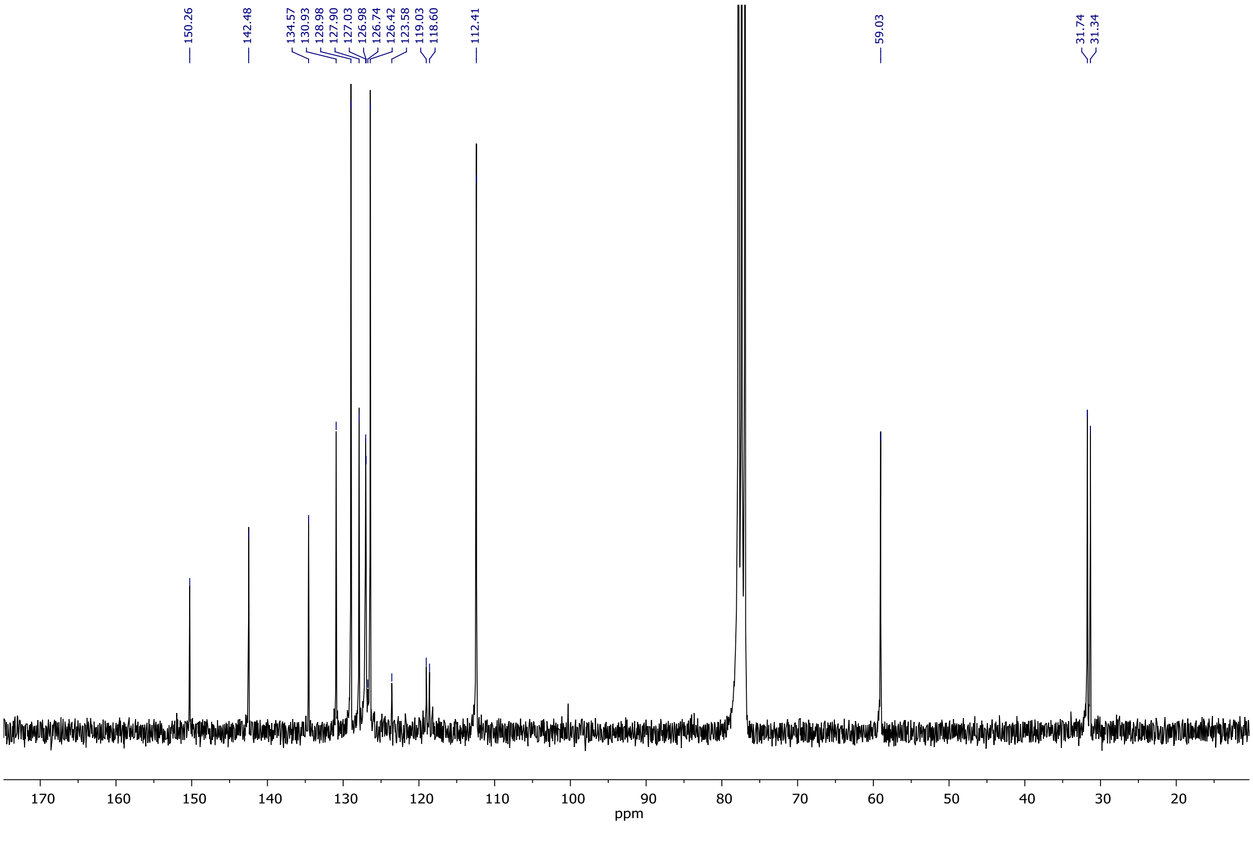
150.26
142.48
134.57
130.93
128.98
127.90
127.03
126.98
126.74
126.42
123.58
119.03
118.60
112.41
59.03
31.74
31.34
ppm

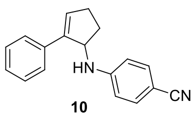
HN
CN

10

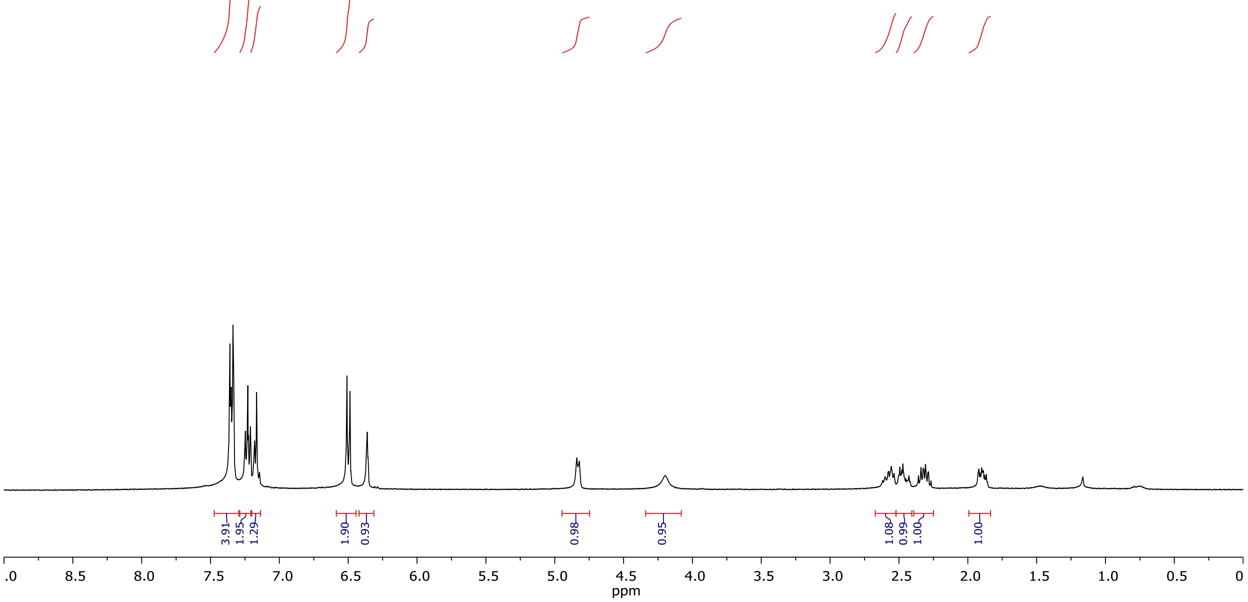
3.91
1.95
1.29
1.90
0.93
0.98
0.95
1.08
0.99
1.00
1.00
ppm

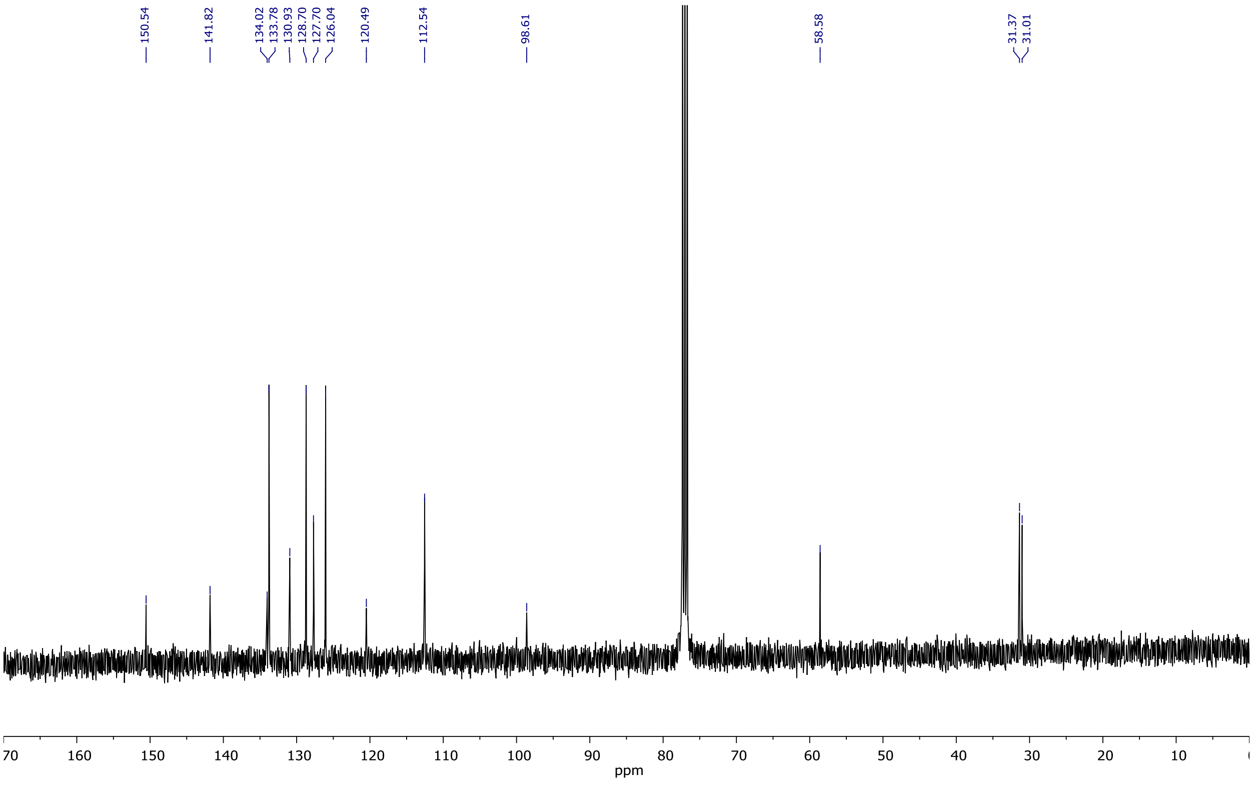
150.54
141.82
134.02
133.78
130.93
128.70
127.70
126.04
120.49
112.54
98.61
58.58
31.37
31.01
ppm

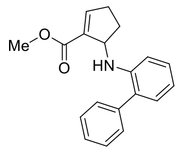

Me
O
O
HN

11

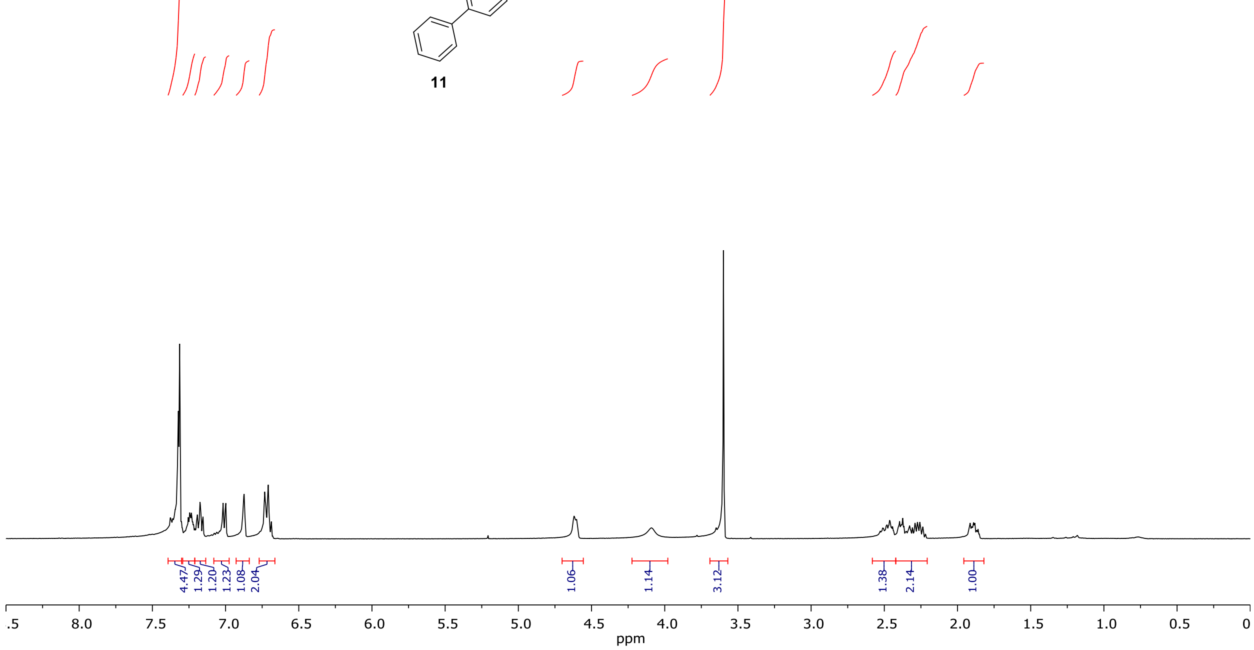

4.47
1.29
1.20
1.23
1.08
2.04
1.06
1.14
3.12
1.38
2.14
1.00
.5
8.0
7.5
7.0
6.5
6.0
5.5
5.0
4.5
4.0
3.5
3.0
2.5
2.0
1.5
1.0
0.5
0
ppm

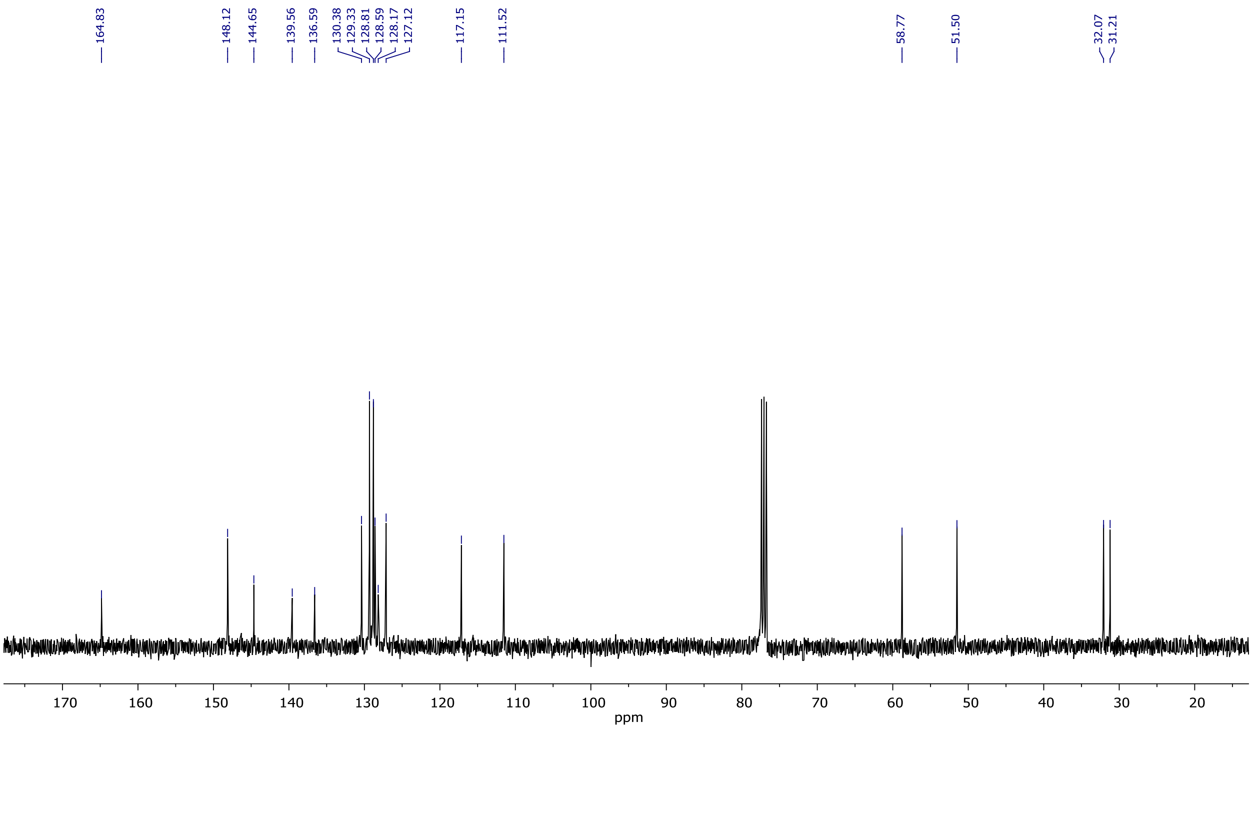

164.83
148.12
144.65
139.56
136.59
130.38
129.33
128.81
128.59
128.17
127.12
117.15
111.52
58.77
51.50
32.07
31.21
170
160
150
140
130
120
110
100
90
80
70
60
50
40
30
20
ppm

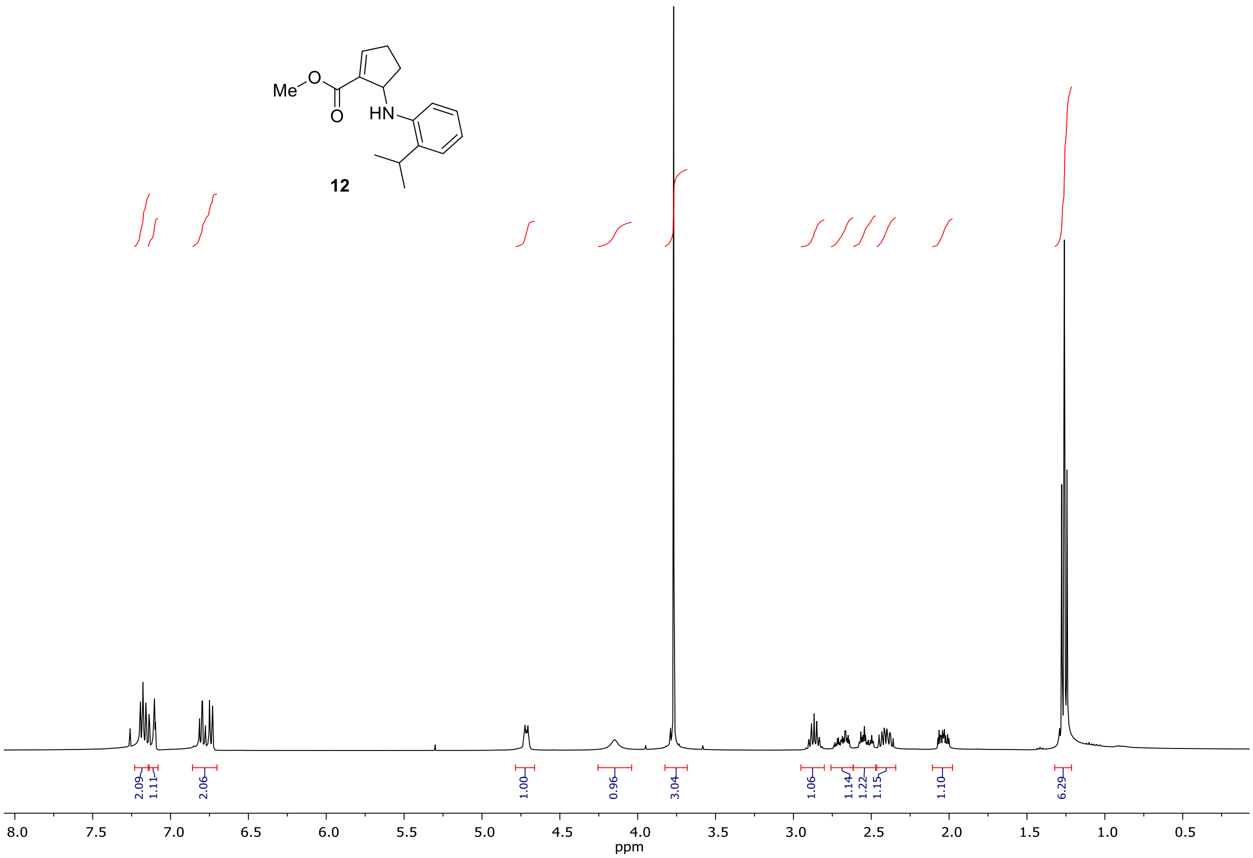
Me
O
O
HN
12
2.09
1.11
2.06
1.00
0.96
3.04
1.06
1.14
1.22
1.15
1.10
6.29
8.0
7.5
7.0
6.5
6.0
5.5
5.0
4.5
4.0
3.5
3.0
2.5
2.0
1.5
1.0
0.5
ppm

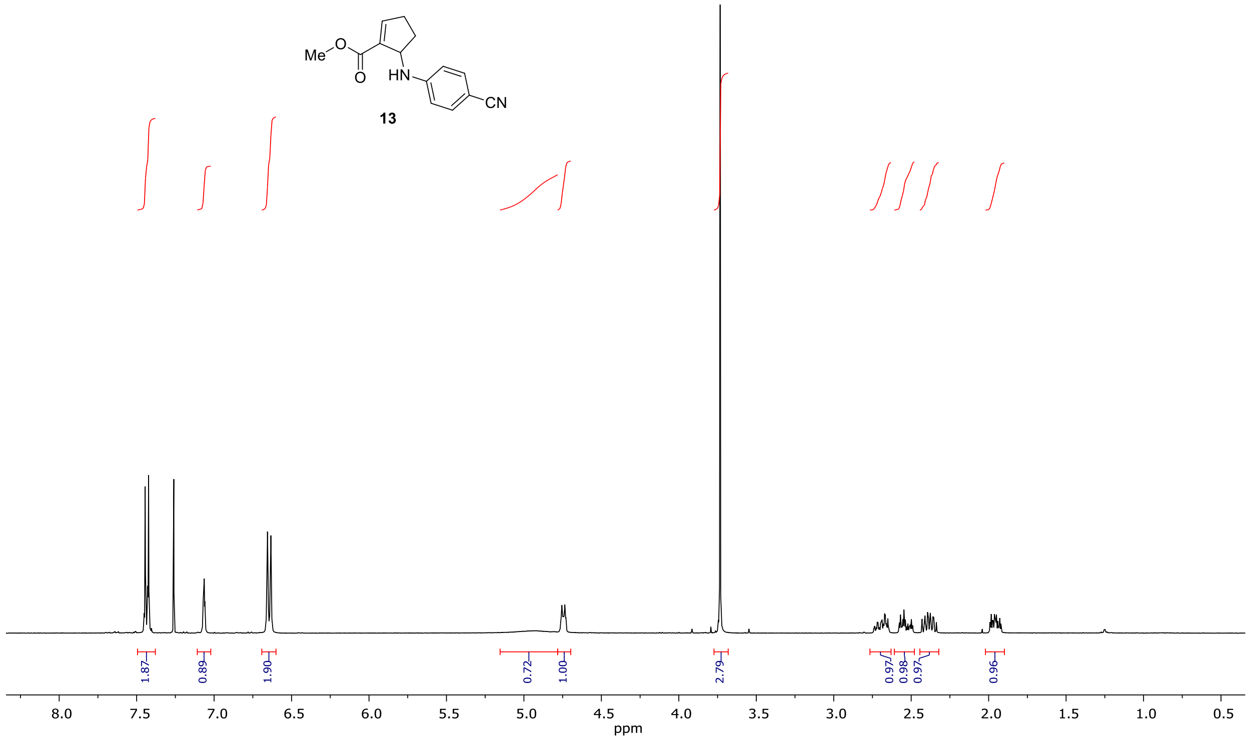
Me
O
O
HN
CN
13
1.87
0.89
1.90
0.72
1.00
2.79
0.97
0.98
0.97
0.96
8.0
7.5
7.0
6.5
6.0
5.5
5.0
4.5
4.0
3.5
3.0
2.5
2.0
1.5
1.0
0.5
ppm

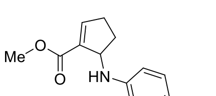
Me
O
O
HN
CF_3

14

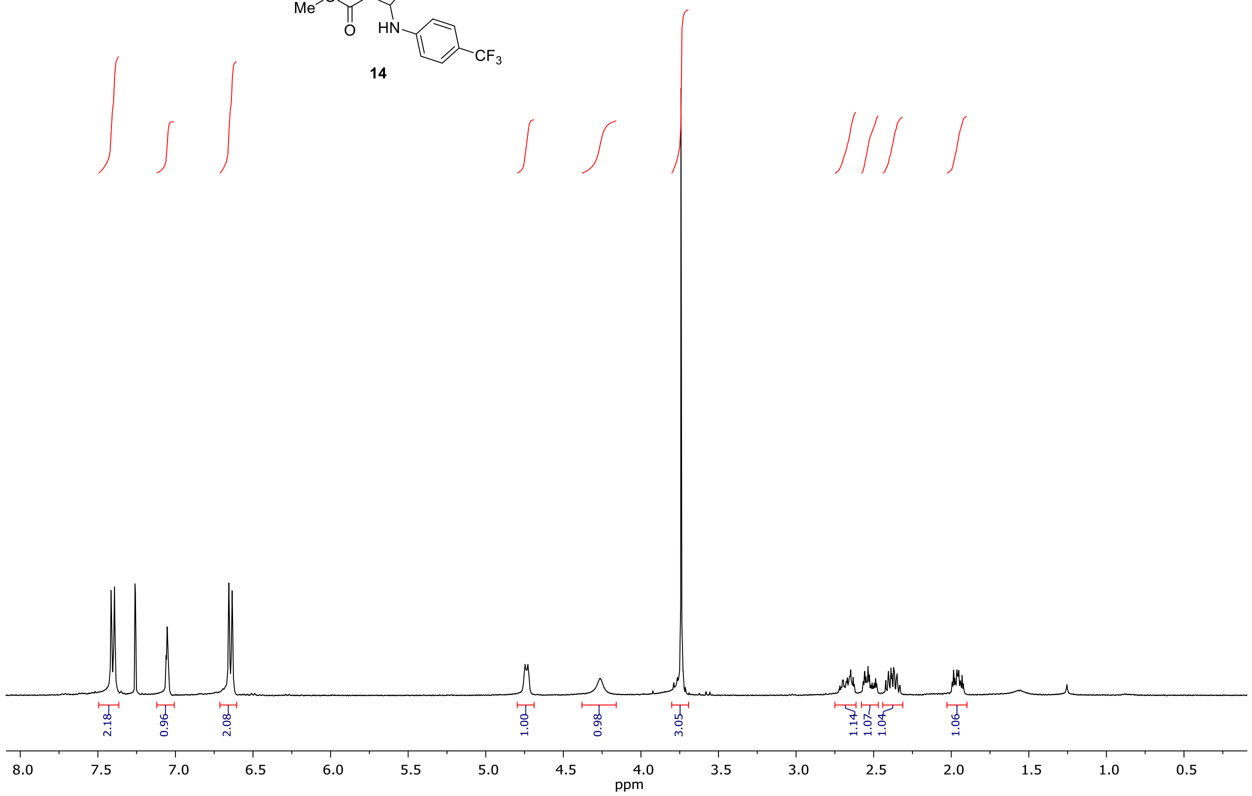
2.18
0.96
2.08
1.00
0.98
3.05
1.14
1.07
1.04
1.06
8.0 7.5 7.0 6.5 6.0 5.5 5.0 4.5 4.0 3.5 3.0 2.5 2.0 1.5 1.0 0.5
ppm

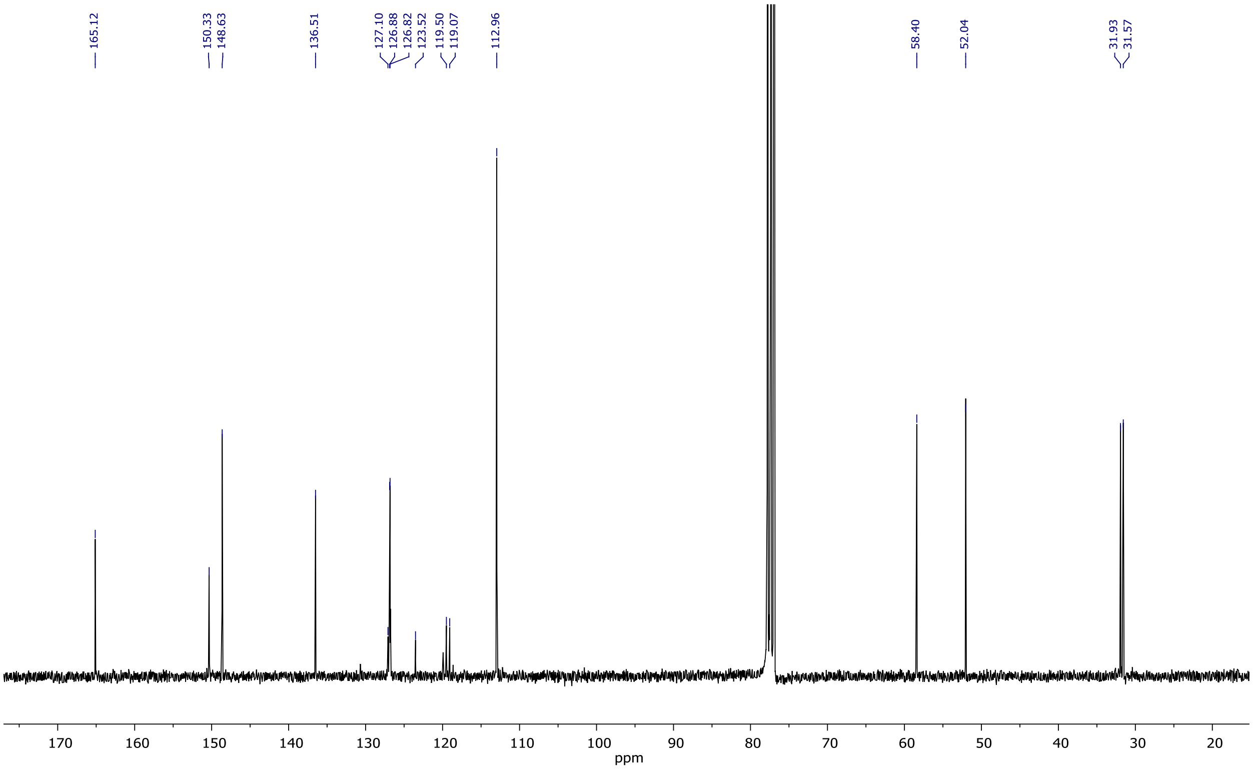
165.12
150.33
148.63
136.51
127.10
126.88
126.82
123.52
119.50
119.07
112.96
58.40
52.04
31.93
31.57
170 160 150 140 130 120 110 100 90 80 70 60 50 40 30 20
ppm

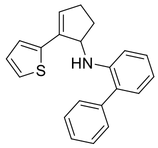

15

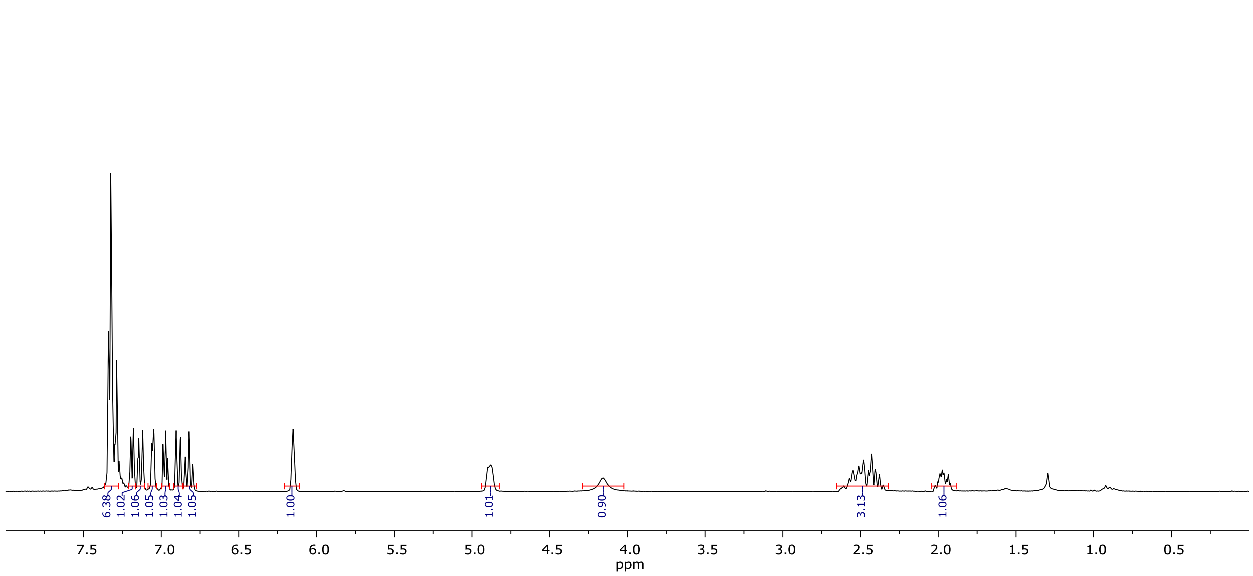
6.38
1.02
1.06
1.05
1.05
1.03
1.04
1.05
1.00
1.01
0.90
3.13
1.06
7.5 7.0 6.5 6.0 5.5 5.0 4.5 4.0 3.5 3.0 2.5 2.0 1.5 1.0 0.5
ppm

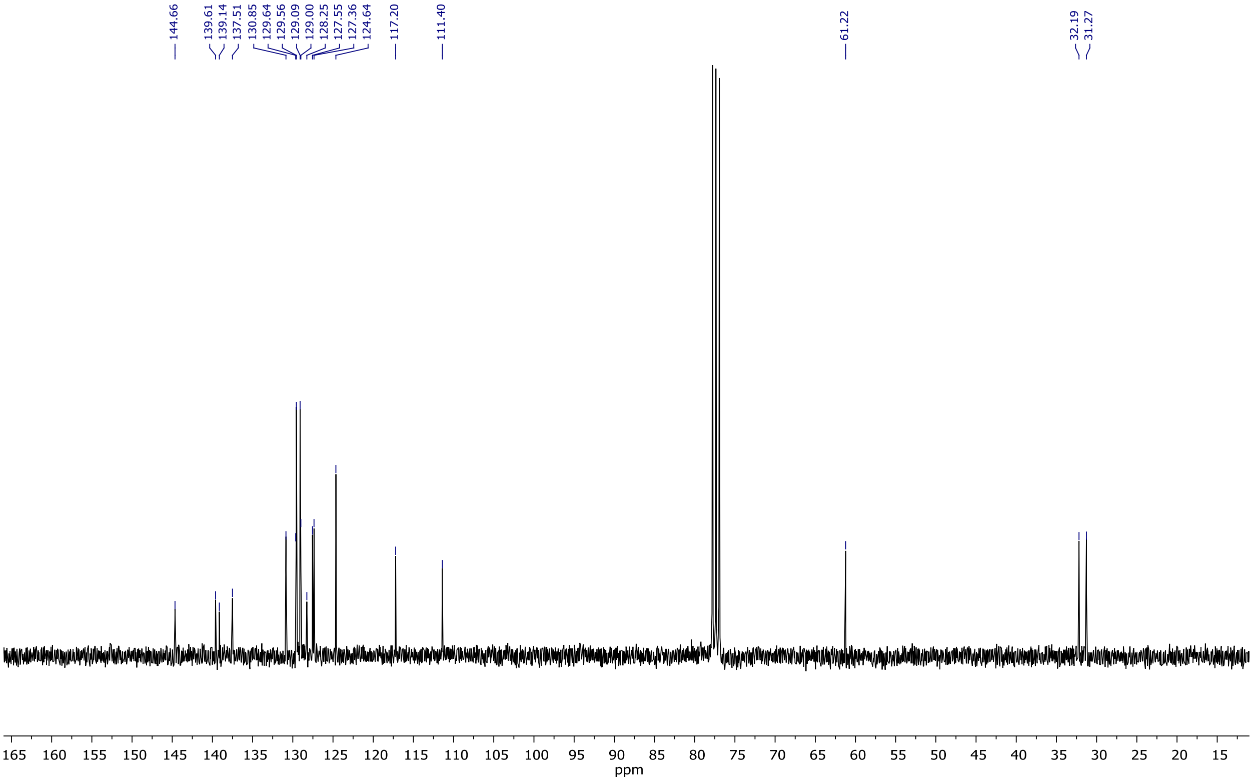
144.66
139.61
139.14
137.51
130.85
129.64
129.56
129.09
129.00
128.25
127.55
127.36
124.64
117.20
111.40
61.22
32.19
31.27
165 160 155 150 145 140 135 130 125 120 115 110 105 100 95 90 85 80 75 70 65 60 55 50 45 40 35 30 25 20 15
ppm

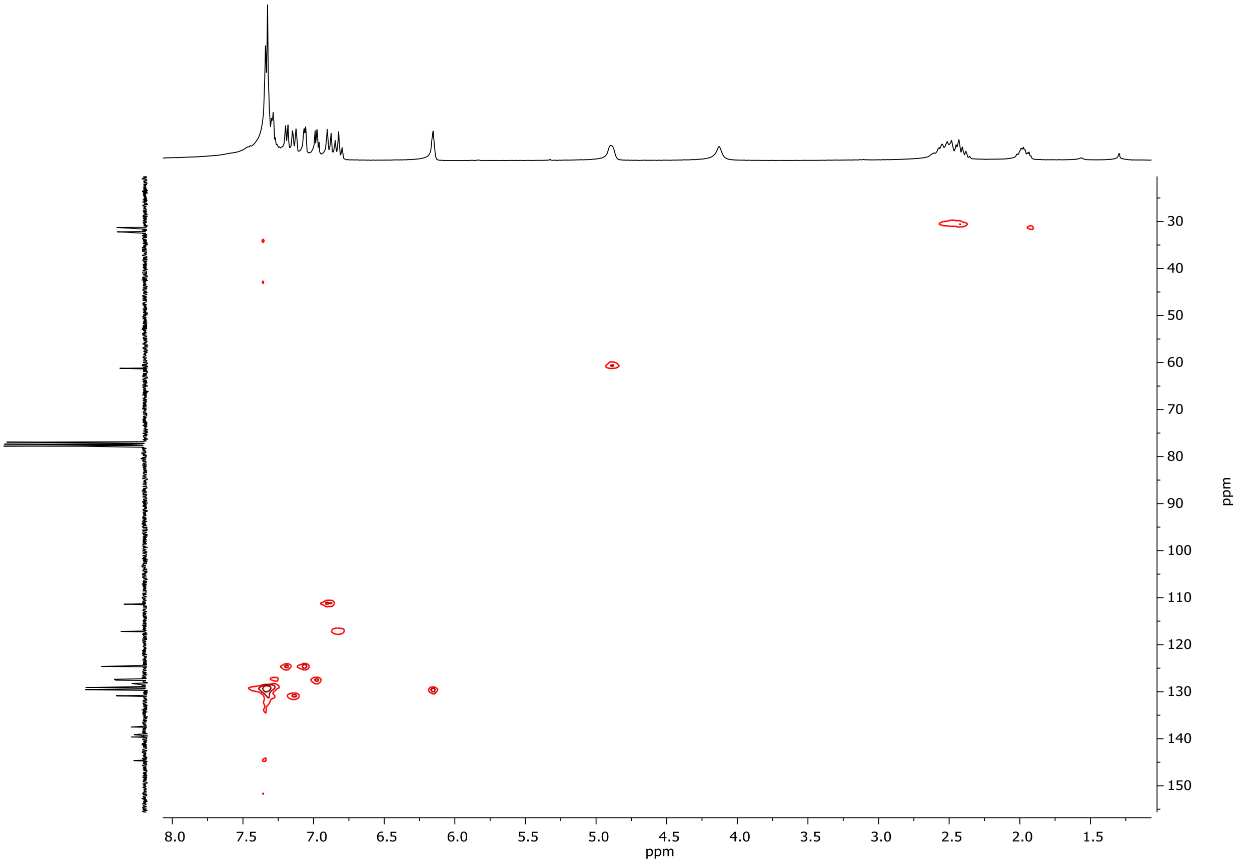
ppm
ppm

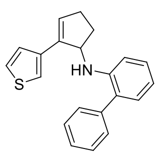
S
HN

16

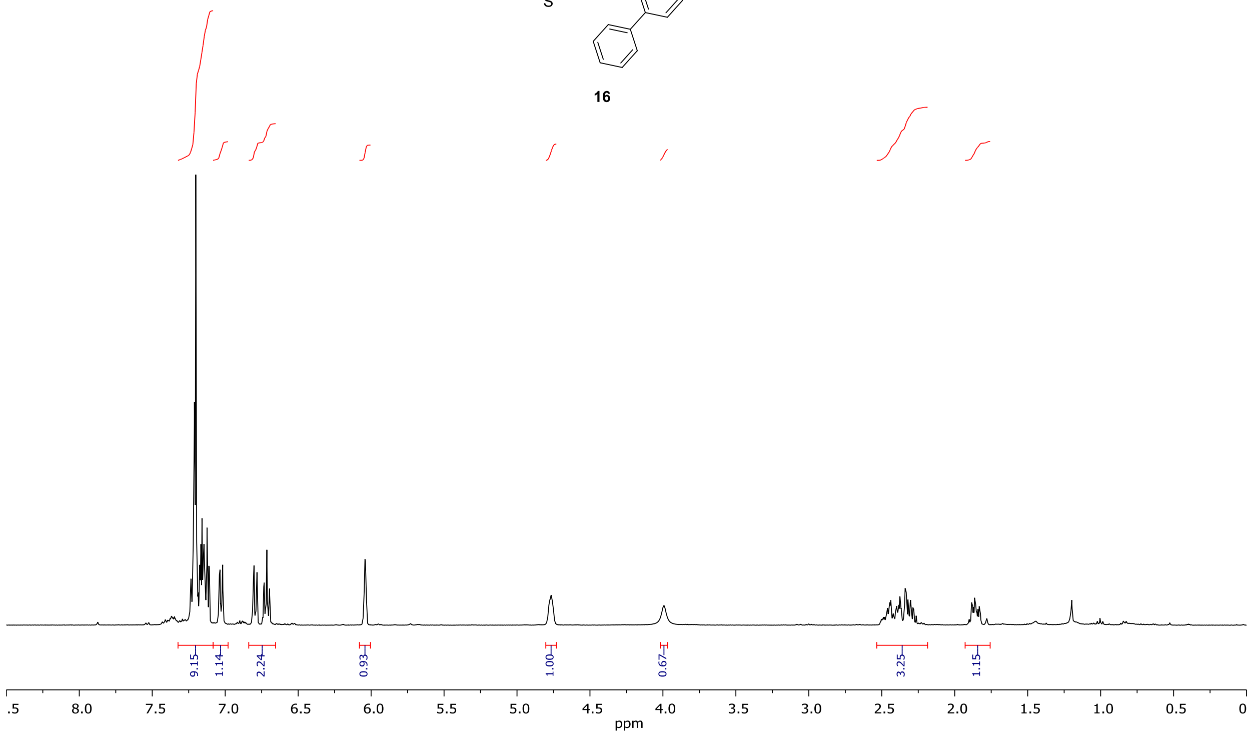
9.15
1.14
2.24
0.93
1.00
0.67
3.25
1.15
.5 8.0 7.5 7.0 6.5 6.0 5.5 5.0 4.5 4.0 3.5 3.0 2.5 2.0 1.5 1.0 0.5 0
ppm

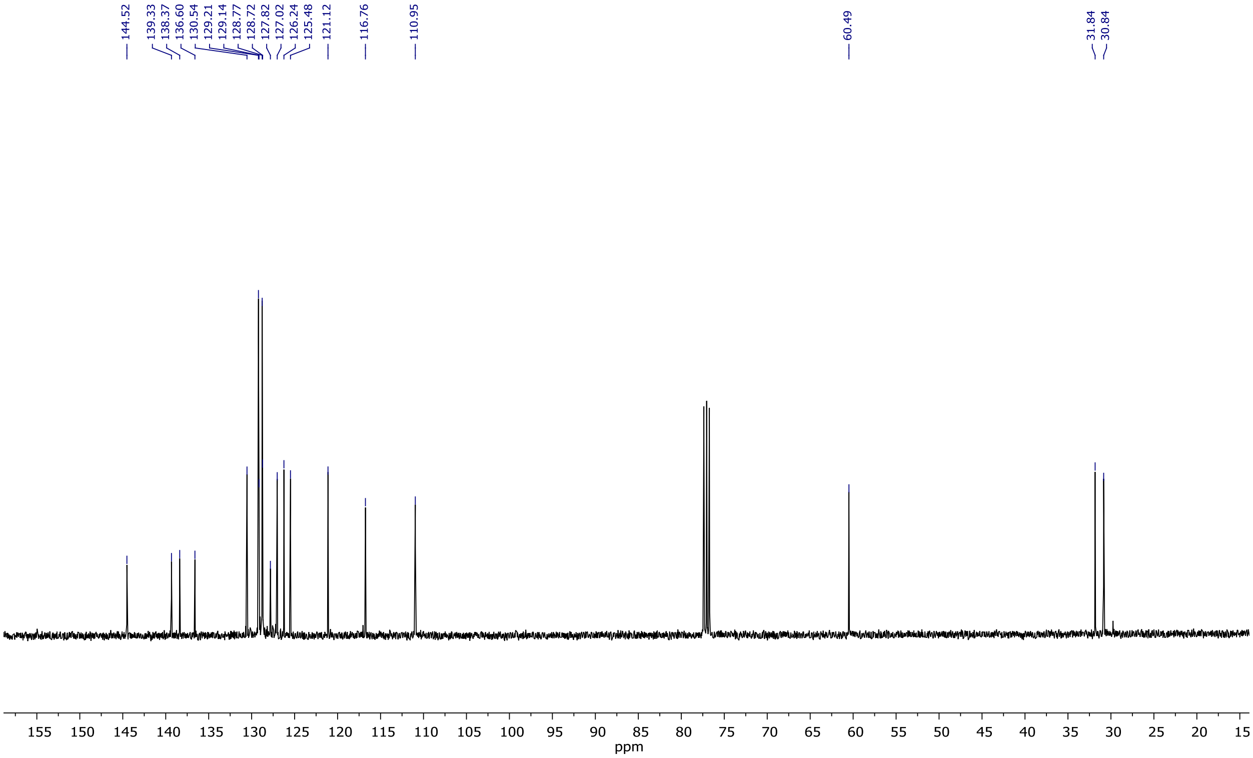
144.52
139.33
138.37
136.60
130.54
129.21
129.14
128.77
128.72
127.82
127.02
126.24
125.48
121.12
116.76
110.95
60.49
31.84
30.84
155 150 145 140 135 130 125 120 115 110 105 100 95 90 85 80 75 70 65 60 55 50 45 40 35 30 25 20 15
ppm

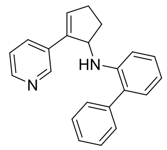
HN
N

17

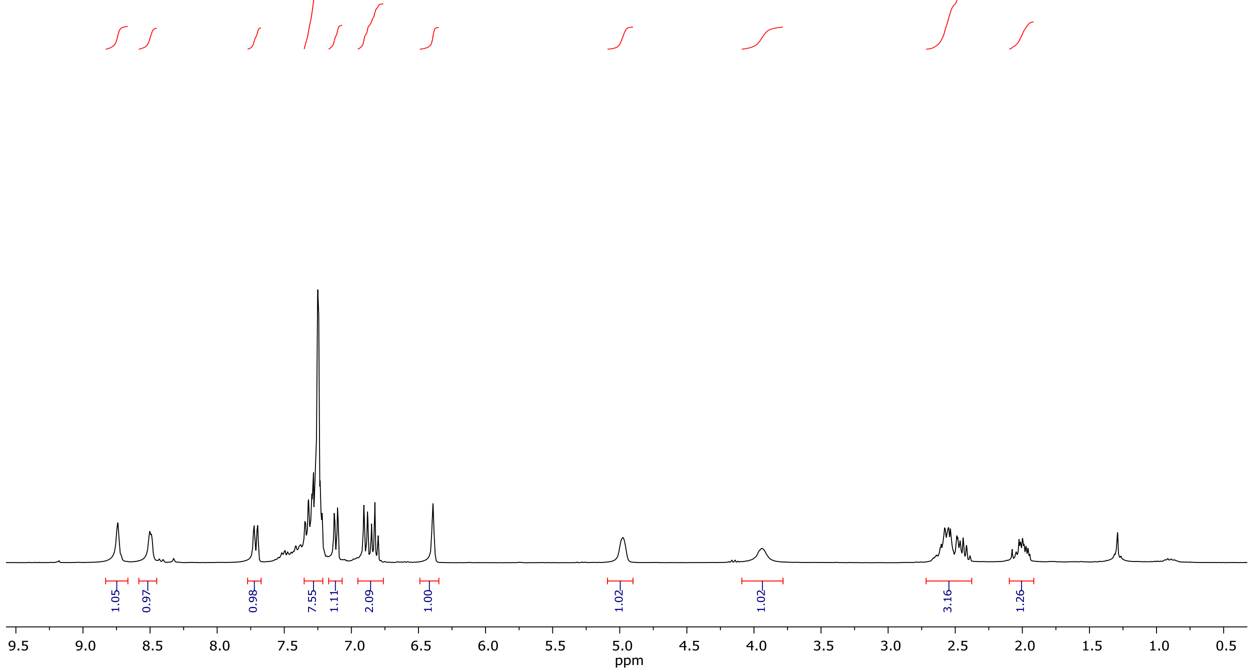
1.05
0.97
0.98
7.55
1.11
2.09
1.00
1.02
1.02
3.16
1.26
9.5 9.0 8.5 8.0 7.5 7.0 6.5 6.0 5.5 5.0 4.5 4.0 3.5 3.0 2.5 2.0 1.5 1.0 0.5
ppm

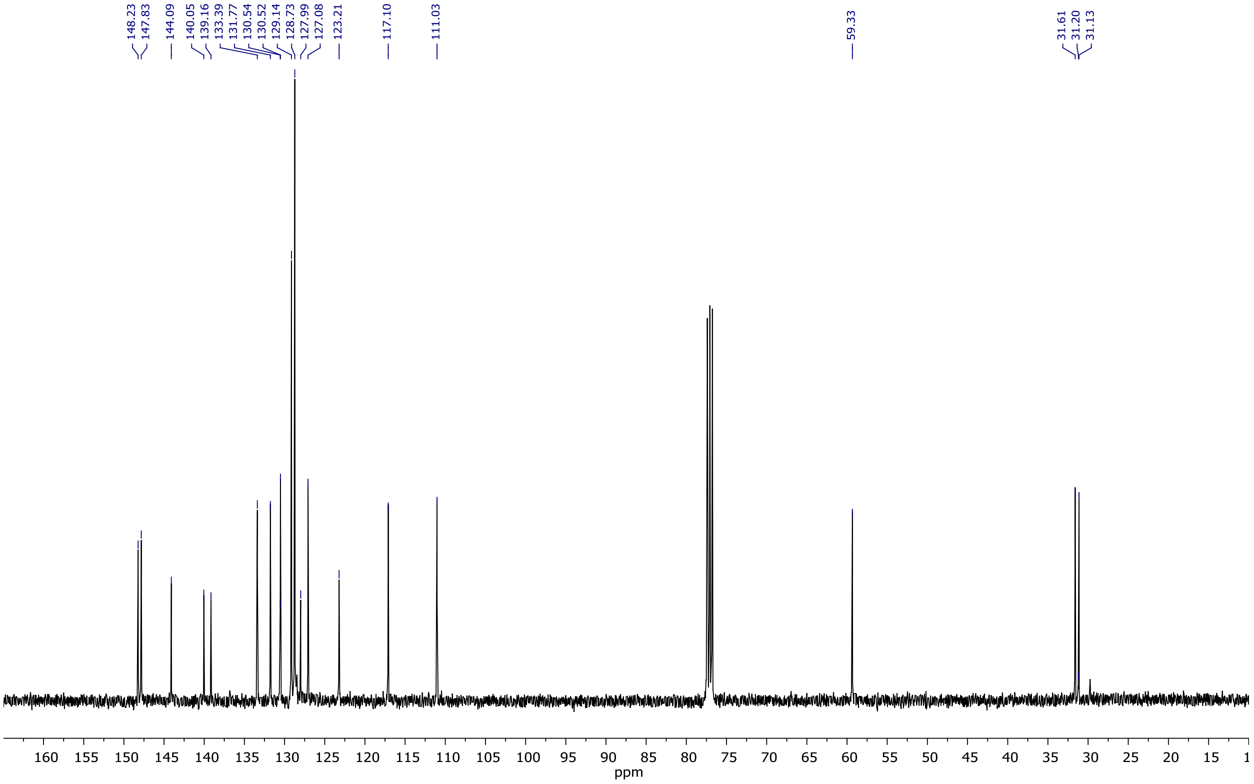
148.23
147.83
144.09
140.05
139.16
133.39
131.77
130.54
130.52
129.14
128.73
127.99
127.08
123.21
117.10
111.03
59.33
31.61
31.20
31.13
160 155 150 145 140 135 130 125 120 115 110 105 100 95 90 85 80 75 70 65 60 55 50 45 40 35 30 25 20 15 1
ppm

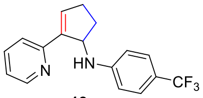

N
HN
CF_3

18

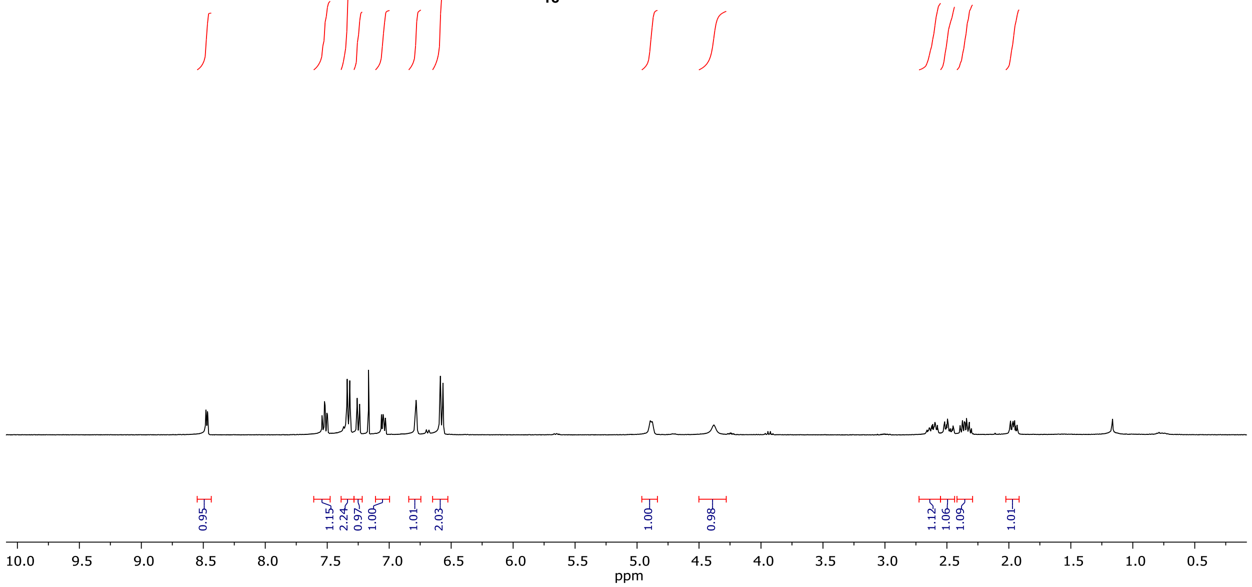

0.95
1.15
2.24
0.97
1.00
1.01
2.03
1.00
0.98
1.12
1.06
1.09
1.01
10.0 9.5 9.0 8.5 8.0 7.5 7.0 6.5 6.0 5.5 5.0 4.5 4.0 3.5 3.0 2.5 2.0 1.5 1.0 0.5
ppm

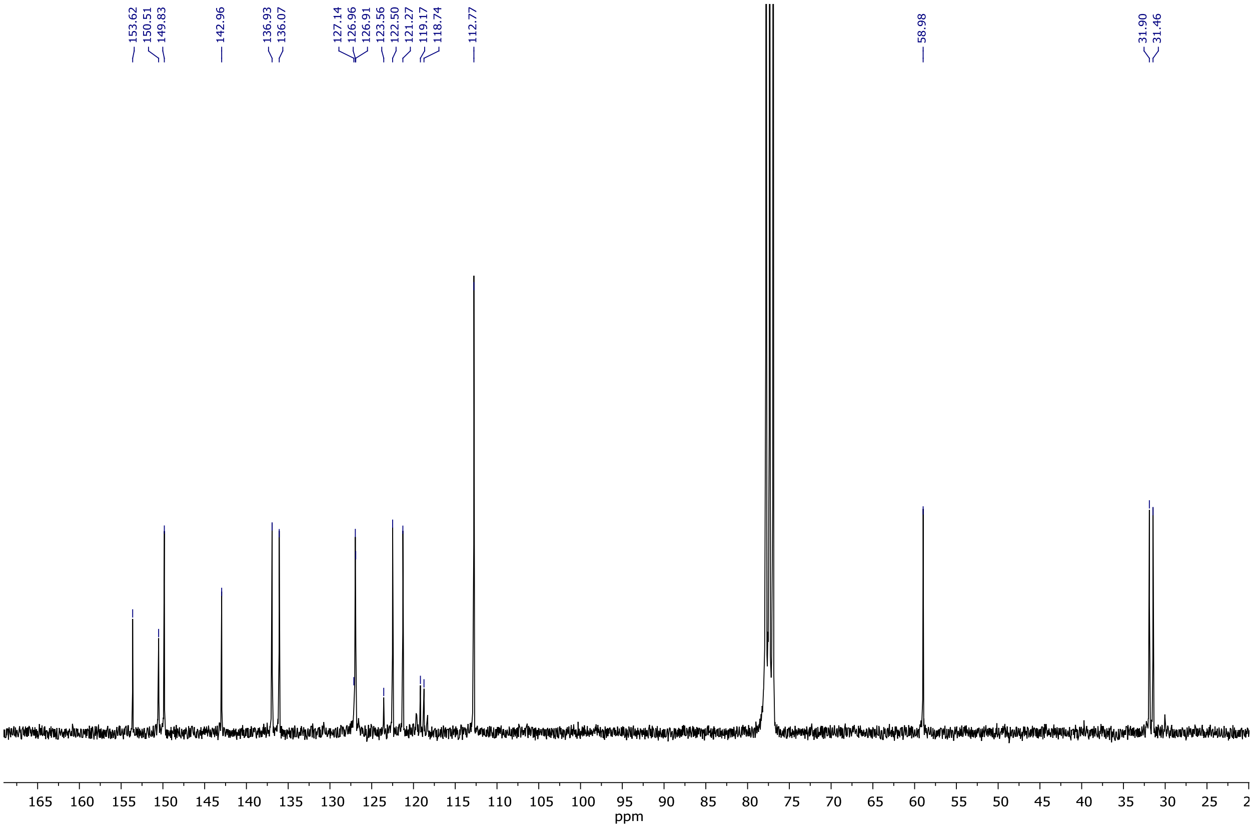

153.62
150.51
149.83
142.96
136.93
136.07
127.14
126.96
126.91
123.56
122.50
121.27
119.17
118.74
112.77
58.98
31.90
31.46
165 160 155 150 145 140 135 130 125 120 115 110 105 100 95 90 85 80 75 70 65 60 55 50 45 40 35 30 25 2
ppm

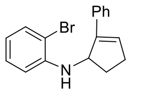
Ph
Br
N
H

21

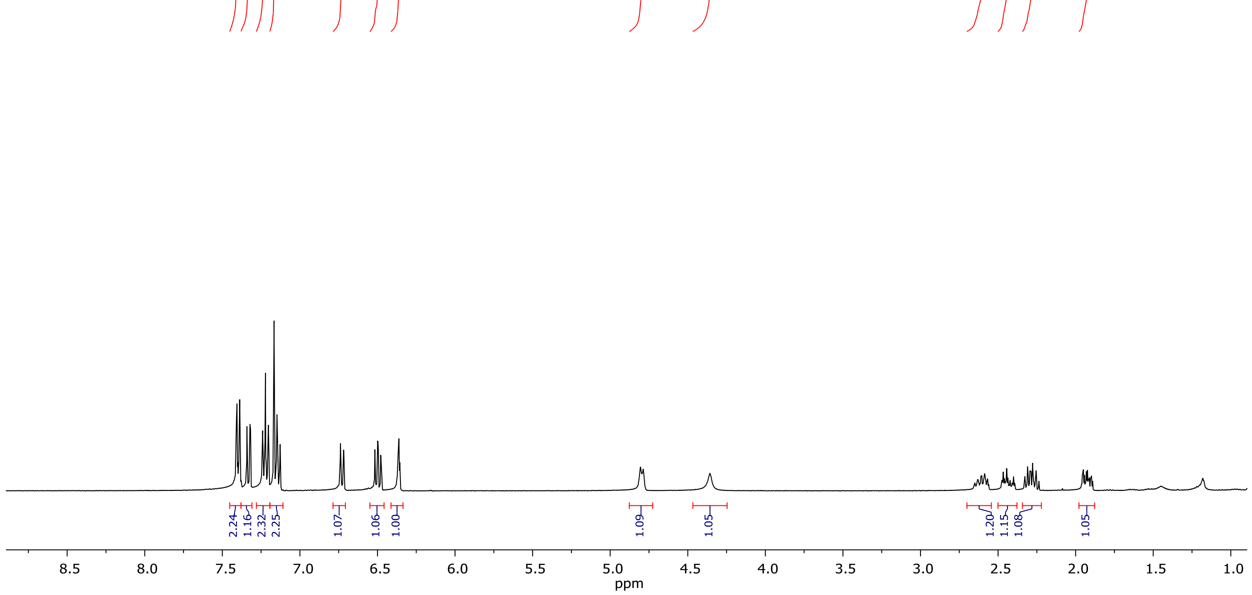
2.24
1.16
2.32
2.25
1.07
1.06
1.00
1.09
1.05
1.20
1.15
1.08
1.05
8.5 8.0 7.5 7.0 6.5 6.0 5.5 5.0 4.5 4.0 3.5 3.0 2.5 2.0 1.5 1.0
ppm

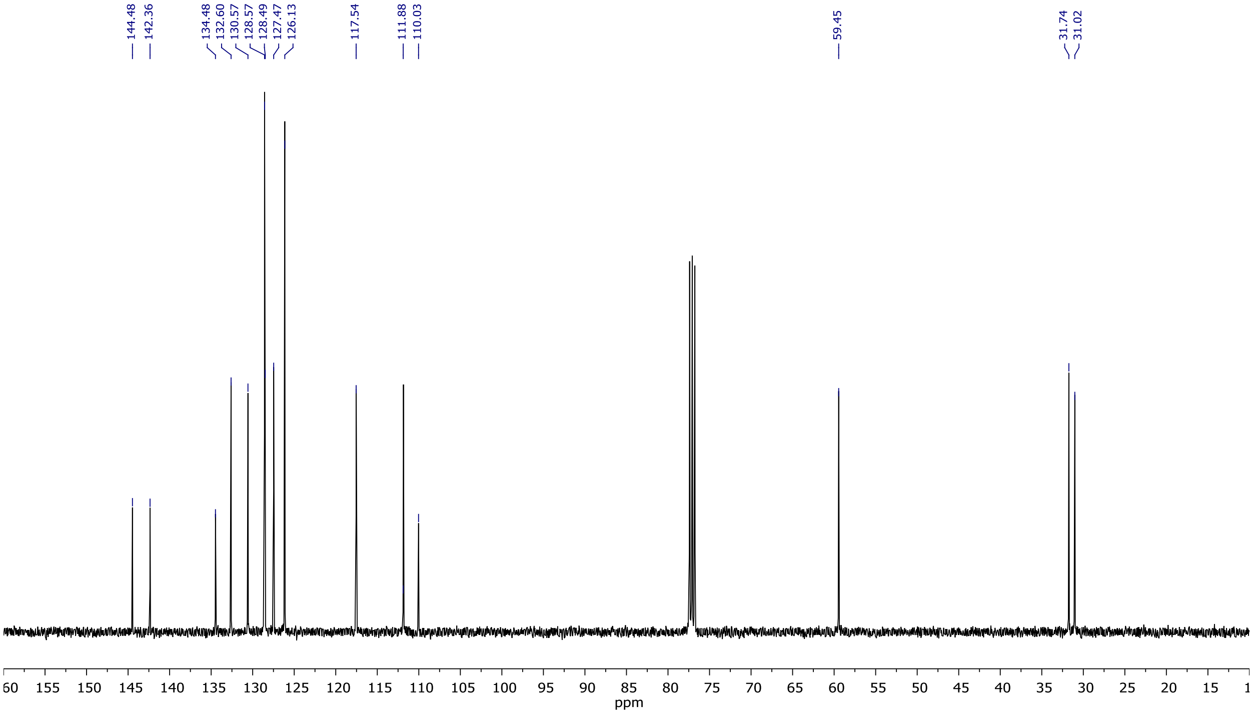
144.48
142.36
134.48
132.60
130.57
128.57
128.49
127.47
126.13
117.54
111.88
110.03
59.45
31.74
31.02
155 150 145 140 135 130 125 120 115 110 105 100 95 90 85 80 75 70 65 60 55 50 45 40 35 30 25 20 15
ppm

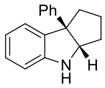
Ph
N
H
H

23

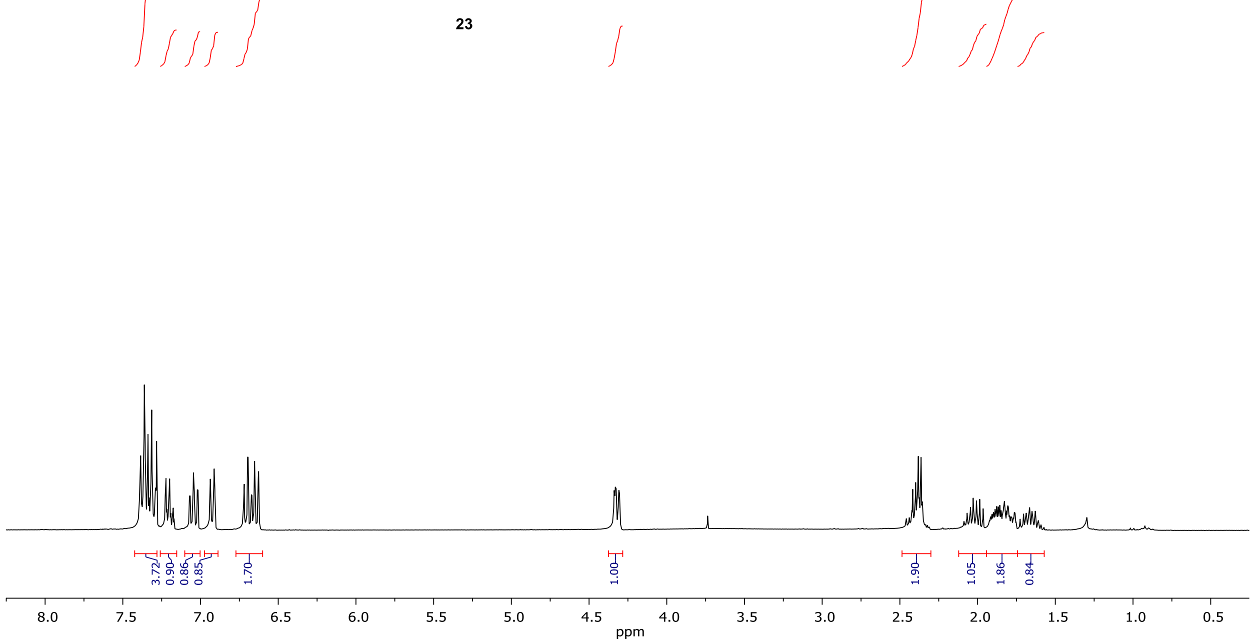
3.72
0.90
0.86
0.85
1.70
1.00
1.90
1.05
1.86
0.84
8.0 7.5 7.0 6.5 6.0 5.5 5.0 4.5 4.0 3.5 3.0 2.5 2.0 1.5 1.0 0.5
ppm

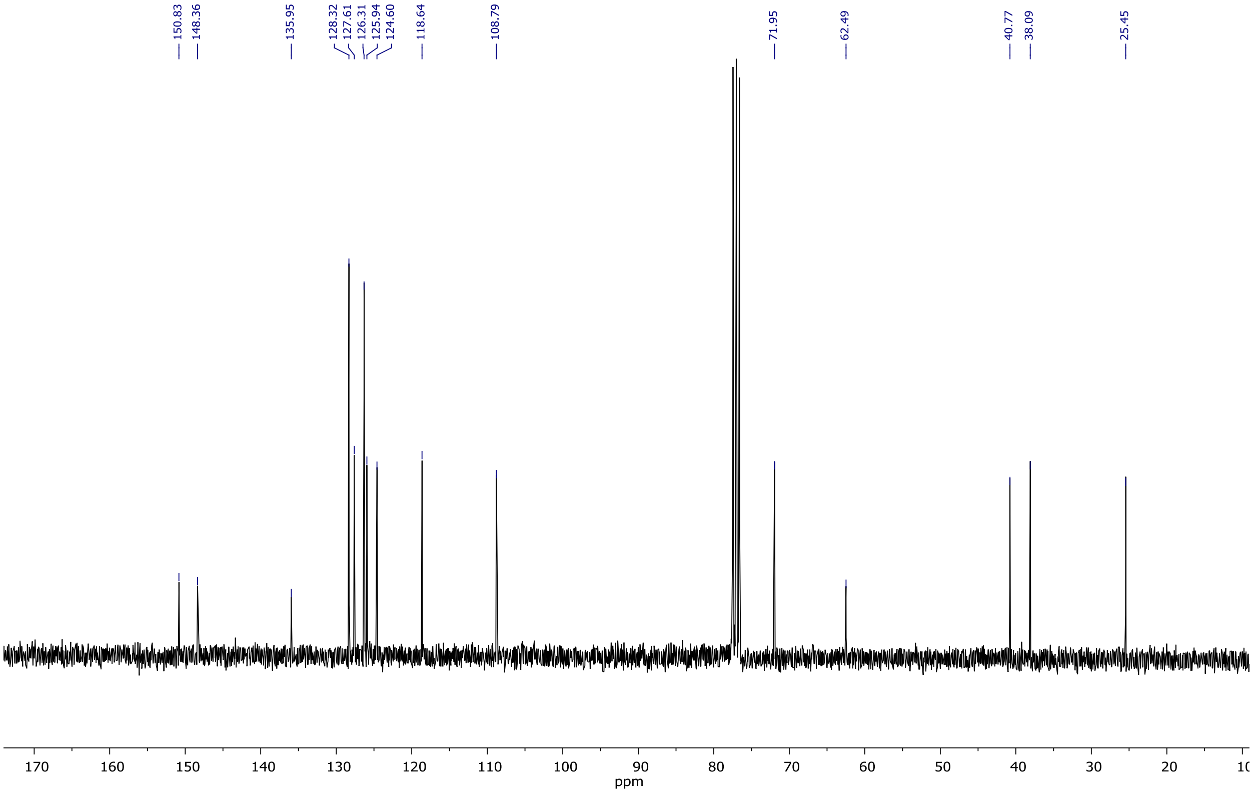
150.83
148.36
135.95
128.32
127.61
126.31
125.94
124.60
118.64
108.79
71.95
62.49
40.77
38.09
25.45
170 160 150 140 130 120 110 100 90 80 70 60 50 40 30 20
ppm
